# ACBD3 modulates KDEL receptor interaction with PKA for its trafficking via tubulovesicular carrier

**DOI:** 10.1186/s12915-021-01137-7

**Published:** 2021-09-07

**Authors:** Xihua Yue, Yi Qian, Lianhui Zhu, Bopil Gim, Mengjing Bao, Jie Jia, Shuaiyang Jing, Yijing Wang, Chuanting Tan, Francesca Bottanelli, Pascal Ziltener, Sunkyu Choi, Piliang Hao, Intaek Lee

**Affiliations:** 1 School of Life Science and Technology, ShanghaiTech University, Pudong, Shanghai, China; 2 School of Physical Science and Technology, ShanghaiTech University, Pudong, Shanghai, China; 3 University of Chinese Academy of Sciences, Beijing, China; 4 Institut für Biochemie, Freie Universität Berlin, Thielallee 63, 14195 Berlin, Germany; 5 Department of Cell Biology, Yale University School of Medicine, New Haven, CT USA; 6 Proteomics Core, Weill Cornell Medicine-Qatar, Doha, Qatar; 7 Shanghai Institute for Advanced Immunochemical Studies, Shanghai, China

**Keywords:** KDEL receptor, Protein Kinase A, ACBD3, ArfGAPs, Arf1-GTP, Golgi

## Abstract

**Background:**

KDEL receptor helps establish cellular equilibrium in the early secretory pathway by recycling leaked ER-chaperones to the ER during secretion of newly synthesized proteins. Studies have also shown that KDEL receptor may function as a signaling protein that orchestrates membrane flux through the secretory pathway. We have recently shown that KDEL receptor is also a cell surface receptor, which undergoes highly complex itinerary between trans-Golgi network and the plasma membranes via clathrin-mediated transport carriers. Ironically, however, it is still largely unknown how KDEL receptor is distributed to the Golgi at steady state, since its initial discovery in late 1980s.

**Results:**

We used a proximity-based in vivo tagging strategy to further dissect mechanisms of KDEL receptor trafficking. Our new results reveal that ACBD3 may be a key protein that regulates KDEL receptor trafficking via modulation of Arf1-dependent tubule formation. We demonstrate that ACBD3 directly interact with KDEL receptor and form a functionally distinct protein complex in ArfGAPs-independent manner. Depletion of ACBD3 results in re-localization of KDEL receptor to the ER by inducing accelerated retrograde trafficking of KDEL receptor. Importantly, this is caused by specifically altering KDEL receptor interaction with Protein Kinase A and Arf1/ArfGAP1, eventually leading to increased Arf1-GTP-dependent tubular carrier formation at the Golgi.

**Conclusions:**

These results suggest that ACBD3 may function as a negative regulator of PKA activity on KDEL receptor, thereby restricting its retrograde trafficking in the absence of KDEL ligand binding. Since ACBD3 was originally identified as PAP7, a PBR/PKA-interacting protein at the Golgi/mitochondria, we propose that Golgi-localization of KDEL receptor is likely to be controlled by its interaction with ACBD3/PKA complex at steady state, providing a novel insight for establishment of cellular homeostasis in the early secretory pathway.

**Supplementary Information:**

The online version contains supplementary material available at 10.1186/s12915-021-01137-7.

## Background

Newly synthesized proteins in the endoplasmic reticulum (ER) are transported to the Golgi apparatus for further post-translational modifications and assembly, prior to secretion or sorting into specific subcellular compartments. In the process of this coordinated transport and sorting, highly abundant ER resident chaperones (i.e., BiP and calreticulin) are leaked to the Golgi and subsequently retrieved to the ER by the seven transmembrane KDEL receptors (KDELR) that bind the tetrapeptide “KDEL” motif found in the C-termini of most soluble ER-resident chaperones [1–4]. KDELR play an important role in maintaining homeostasis in the early secretory pathway, as their function helps establish a physiological equilibrium for the core components of protein folding and trafficking [5–7].

KDELR, like the stereotypical seven transmembrane G-protein coupled receptors (GPCRs) family, are suggested to also function as a GPCR-like signaling protein that senses and regulates traffic in the Golgi [6–8], although a recent structural study revealed that KDELR structure more closely resembles SWEET transporter family of proteins, rather than GPCR family of proteins [9].

KDELR are largely concentrated at the Golgi [10, 11], but how this steady-state localization is achieved under physiological condition has remained largely unknown since initial identification of the receptor in the 1980s. The prevalent model suggests that steady state localization of KDELR at the Golgi is likely to result from a dynamic balance between anterograde and retrograde transport of the receptor between the ER and the Golgi without a special retention mechanism [3].

Recent studies have also shown that a small fraction of KDELR may reside at the plasma membrane (PM) and function as a putative cell surface receptor for certain growth factors, although the precise trafficking itinerary and mechanism of KDELR expression at the cell surface is still unclear. We recently reported that KDELR constantly cycles between the PM and the Golgi via Rab14- and Rab11-positive recycling endosomes and that both its endocytosis from the PM and its export from the TGN appear to be mediated by clathrin-coated transport carriers [12].

In this study, we sought to further dissect fundamental mechanisms and molecular components that govern KDEL receptor localization and trafficking at the Golgi. To this end, an in vivo tagging and proteomics method was used to obtain an unbiased KDEL receptor “interactome”. The candidate proteins identified in the proteomic experiments were then tested for their function in localization of KDELR by RNAi-mediated knockdown and confocal microscopy.

We found that ACBD3, which have been associated with highly diverse roles, ranging from asymmetric cell division, the cellular stress response, and bacterial and viral replication at the Golgi [13–17], regulates Golgi localization of all isoforms of KDELR. Yeast two-hybrid experiments and split-YFP assays indicated that KDELR and ACBD3 may interact in a very close proximity. ACBD3 depletion caused a significantly accelerated retrograde trafficking of the receptor to the ER without introduction of massive cargo wave, leading to re-distribution of KDELR to the ER at steady state. Further, ACBD3 appears to influence KDELR trafficking by altering KDELR interaction with Protein Kinase A (PKA) and ArfGAP1/Arf1-dependent tubulovesicular carrier formation at the Golgi. These results reveal an important mechanistic underpinning for KDELR-mediated maintenance of cellular homeostasis in the early secretory pathway.

## Results

### Proximity-based profiling of KDELR-associated proteins reveals ACBD3 and ArfGAP3 as novel interacting partners

**Fig. 1:**
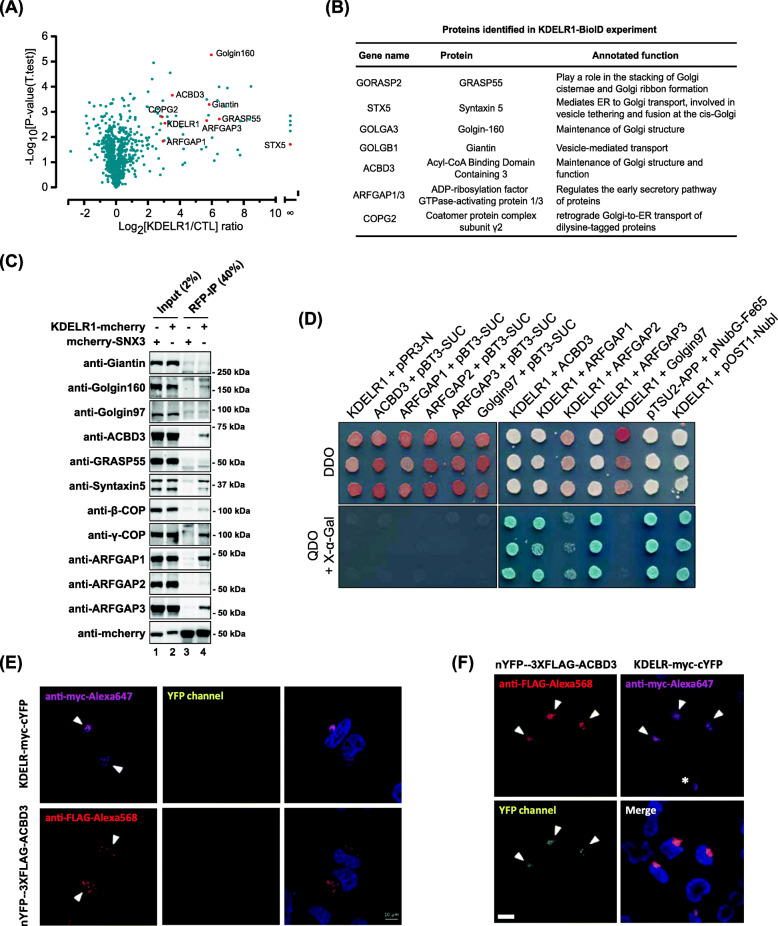
ACBD3 is a novel binding partner of KDELR1. **A** Volcano plot representing results of the label-free BioID of KDELR1. The logarithmic ratio of protein intensities in the KDELR1-BioID/KDELR1-myc were plotted against negative logarithmic *p*-values of the *t* test performed from the triplicates. Annotated Golgi proteins are candidate interaction partners of KDELR1 with a *p*-value of ratio significance <0.05 and marked with red dots. **B** Table summarizing annotated function of the Golgi proteins identified in KDELR1-BioID experiments. **C** Several Golgi-related proteins co-immunoprecipitate with KDELR1-mCherry. The protein extracts from HeLa cells transfected with mCherry tagged Sorting Nexin 3 (mCherry-SNX3 as a control) or KDELR1-mCherry were immunoprecipitated with anti-RFP agarose beads. These lysates and the immunoprecipitates (anti-RFP IPs) were analyzed by western blotting using anti-mCherry antibody and antibodies against the indicated Golgi proteins. **D** Split-ubiquitin-based membrane yeast two-hybrid assays suggest that KDELR1 interact with ACBD3 and ARFGAP1/3, but not with ARFGAP2 and Golgin97. Growth of yeast cells expressing KDELR1-Cub-LexA-VP16 bait or a pBT3-SUC bait vector with the following NubG-HA-preys: ACBD3, ARFGAP1, ARFGAP2, ARFGAP3, Golgin97, pPR3-N (prey vector as a negative control), and Ost1-NubI (positive control) were challenged on agar plates depleted of tryptophan and leucine (DDO plates, upper panel) or depleted of tryptophan, leucine, histidine, and adenine (QDO plates, bottom panel), plus 40 μg/ml X-α-Gal by spotting three independent transformants on different plates. The bait pTSU2-APP and the prey pNubG-Fe65 were used as positive controls in the MYTH assay. Experiments were repeated twice. **D**, **E** Interaction between ACBD3 and KDELR1 detected by split-YFP assays. HeLa cells were transfected with nYFP-3xFLAG-ACBD3 and KDELR1-myc-cYFP for 18 h before cells were fixed and observed by confocal microscopy (**E**). Single plasmid (nYFP-3xFLAG-ACBD3 or KDELR1-myc-cYFP) transfection was used as negative controls (**D**). White arrowheads indicate co-transfected cells, whereas white asterisks indicate cells transfected with a single plasmid, serving as a negative control. Scale bars = 10 μm

To obtain KDELR interactome, we used a recently developed proteomics technique called “BioID” [18, 19], in which proximate and interacting proteins are tagged in living cells, prior to cell disruption and analysis of the tagged proteins by mass spectrometry. We fused human KDELR1 (Genebank: AAH18778.1) at its C-terminus to a bacterial biotin ligase BirA with a point mutation (referred as BirA*), as described by Roux et al. [18]. Correct localization of the biotinylated proteins was confirmed by immunofluorescence (Additional file 1: Figure S1A). The construct was then transfected into HeLa cells for 24 h, followed by biotinylation for 6 h in vivo. Cells were then lysed in lysis buffer and tagged proteins were purified by incubation with Streptavidin-agarose. Isolated proteins were then analyzed by mass spectrometry. The results identified a number of Golgi-associated membrane trafficking proteins, such as Giantin, Golgin160, ACBD3, GRASP55, Syntaxin 5, and ArfGAP3, as well as proteins already known to be involved in retrograde transport of KDELR, such as ArfGAP1 [20] and some coatomer subunits [21] (Fig. 1A, B; Additional file 1: Figure S1B-C).

Western blot analysis of the BioID samples was consistent with the mass spec results (Additional file 1: Figure S1C). Co-immunoprecipitation (co-IP) experiments using anti-RFP agarose against KDEL-R1-mCherry further validated ACBD3, Golgin160, ArfGAP1, and ArfGAP3 as interacting partners that may form stable protein complexes with KDELR, whereas Giantin and GRASP55 were not significantly co-immunoprecipitated with KDELR1-mCherry in the same experiments (Fig. 1C). Each of these proteins from BioID results were knocked down using RNA interference to test whether KDELR trafficking is significantly influenced upon their depletion. Unexpectedly, none of the ArfGAPs and Golgin160 showed any significant changes in KDEL1-mCherry localization to the Golgi upon their depletion. Instead, only ACBD3 depletion resulted in significant re-distribution of KDELR1-mCherry to the ER (Additional file 1: Figure S1D-K).

Taken together, these results suggested that ACBD3 is a novel binding protein of KDELR1, which may regulate KDELR trafficking at the Golgi, and that this role is likely to be independent of its interaction with diverse Golgi tethering proteins, including Giantin and Golgin160.

### ACBD3 interacts with KDELR at the Golgi

Since ACBD3 is known as a multi-functional protein [22], it was important to determine whether ACBD3 can interact with KDELR at the Golgi, prior to more in-depth study for the role of ACBD3 in KDELR trafficking and function. To this end, split-ubiquitin-based membrane yeast two-hybrid assays (MYTH) were then used to study whether KDELR1 and ACBD3 may work together in close proximity. For this experiment, KDELR1 served as the “bait” and was tagged at its C terminus with a moiety consisting of Cub (C-terminal fragment of ubiquitin) fused to the transcription factor LexA-VP16, as described in the “Methods” section. ACBD3, ArfGAP1, ArfGAP2, ArfGAP3, and Golgin97 (negative control) were tagged at their N terminus with the NubG (a mutant of N-terminal fragment of ubiquitin). The C-terminal tail of KDELR1 had previously shown to directly interact with ArfGAP1 [21, 23, 24], so ArfGAP1 was included as a positive control in this experiment.

Using this MYTH assay, we found that KDELR1 occurs within molecular contact distance to both ACBD3 and ArfGAP1/3, indicated by the growth of blue yeast colonies expressing KDELR1 with ACBD3 or ArfGAP1/3 on interaction selection medium (Fig. 1D, QDO + X-α-gal panel). In contrast, yeast colonies expressing KDELR1 with ArfGAP2 or Golgin97, KDELR1 with pPR3-N prey vector or preys with pBT3-SUC bait vector grew only on transformation selection medium (Fig. 1D, DDO panel), confirming the specificity of the interactions.

To study the in vivo interaction between KDELR and ACBD3, we used split-YFP assays, in which KDELR was fused to myc-tag, followed by C-terminal half of YFP at its tail (KDELR-myc-cYFP) and ACBD3 was fused to N-terminal half of YFP and 3xFLAG-tag (nYFP-3xFLAG-ACBD3). These two constructs were then individually transfected into HeLa cells to check any background signal in YFP channel of confocal microscope. The results (Fig. 1E) indicated that there was no significant background noise in YFP channel, when either of the two constructs was expressed individually.

We then co-transfected nYFP-3xFLAG-ACBD3 and KDELR-myc-cYFP overnight in HeLa cells, in order to test whether we could detect their interaction via fluorescent signal from combined YFP protein. Strikingly, strong YFP signal was detected only in cells, expressing both nYFP-3xFLAG-ACBD3 and KDELR-myc-cYFP (Fig. 1F; white arrowheads). When only KDELR-myc-cYFP was expressed, we did not observe any YFP signal (Fig. 1F; white asterisk). Taken together, these results strongly indicated that ACBD3 may be a novel binding partner of KDELR at the Golgi.

### ACBD3 regulates Golgi localization of all KDELR isoforms

In ACBD3-depleted cells, a large proportion of KDELR1-mCherry co-localized with Calnexin, an ER marker, suggesting ER-specific re-localization of KDELR1-mCherry in ACBD3-depleted cells, which could be restored by re-expression of a RNAi-resistant form of ACBD3 (Additional file 2: Figure S2A). These results were readily reproduced using human fibrosarcoma HT-1080, suggesting that re-distribution of KDELR1-mCherry to the ER upon ACBD3 depletion is unlikely to be a cell-type specific effect (Additional file 2: Figure S2B-C).

In order to rule out the possibility that mCherry tagging at the C-terminal tail of human KDELR may have altered KDELR trafficking and function, we co-transfected a secretory cargo fused C-terminal KDEL sequence (hGH-GFP-KDEL) with KDELR1-mCherry to see whether excess KDEL ligand in the early secretory pathway can induce re-distribution of KDELR1-mCherry to the ER in HeLa cells. The results showed that expression of hGH-GFP-KDEL (but not of control hGH-GFP) caused re-distribution of KDELR1-mCherry to the ER to a significant extent (Additional file 2: Figure S2D), confirming that ligand binding-induced retrograde trafficking of KDELR1-mCherry is unaffected.

**Fig. 2:**
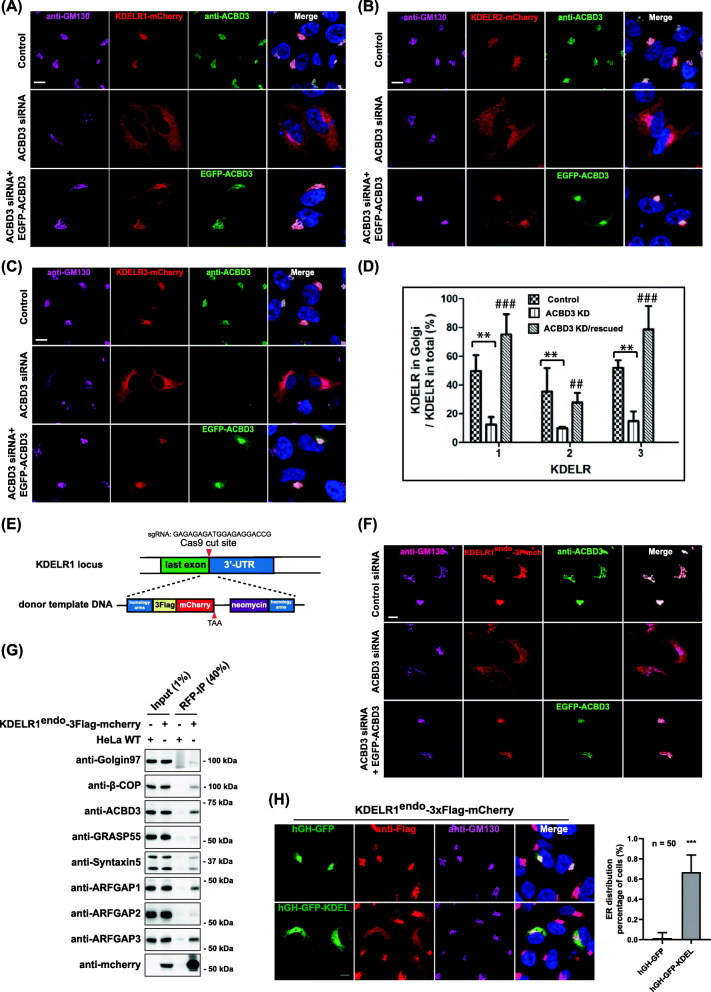
ACBD3 contributes significantly to Golgi localization of KDELR at steady state. **A**–**D** Confocal micrographs of HeLa cells expressing KDELR1-mCherry (**A**), KDELR2-mCherry (**B**), and KDELR3A-mCherry (**C**), showing that depletion of ACBD3 results in significant re-distribution of all three isoforms of KDELRs from the Golgi to the ER in vivo, which could be restored by exogenous expression of RNAi-resistant EGFP-ACBD3. For quantification (**D**), the percentage of fluorescent intensity of Golgi-localized KDELRs over the total KDELR-mCherry were quantified and plotted onto the histogram as average ratio with s.d (*n* = 20 ~ 25) (ACBD3-KD vs Control, **p < 0.001; ACBD3-KD/rescued vs ACBD3-KD, ^##^*p* < 0.01; ^###^*p* < 0.001). **E** Schematic illustration of CRISPR-Cas9-mediated insertion of 3xFlag-mCherry tagging at the C-terminal end of endogenous KDELR1. **F** Confocal micrographs of KDELR1^endo^-3xFlag-mCherry in HeLa cells showing that depletion of ACBD3 results in re-distribution of KDELR1^endo^-3xFlag to the ER in vivo. **G** Endogenously tagged KDELR1-3xFlag-mCherry pulls down ACBD3 and ArfGAP1/3, respectively. HeLa cells with KDELR1-3xFlag-mCherry was lysed and subjected to immunoprecipitation with anti-RFP antibody, followed by western blot analysis. **H** Confocal results showing that hGH-GFP-KDEL expression induces endogenously 3xFLAG-mCherry-tagged KDEL receptor to re-distribute to the ER in HeLa cells. The bar graph to the right summarizes the quantification of these results. (***p < 0.001) Scale bars = 10 μm

We then examined whether ACBD3 depletion results in similar redistribution of other KDEL receptor isoforms. ACBD3 was depleted in HeLa cells by siRNA targeting its 3′UTR for 48 h, and KDELR1, KDELR2, and KDELR3a fused to mCherry at the C-terminus were then transfected for 18 h. The results from confocal experiments showed that all three isoforms of KDELRs were significantly redistributed to the ER in ACBD3-depleted cells (Fig. 2A–D). Upon re-expression of RNAi-resistant EGFP-ACBD3, we observed clear restoration of Golgi localization for all the KDELR isoforms. Quantification of the confocal results indicated that the ratio of Golgi-localized KDELR1- and KDELR3-mCherry to the total amount of red fluorescent signals were reduced by approximately 3-fold in ACBD3-depleted cells, which were completely rescued by re-expression of EGFP-ACBD3.

Furthermore, expression of EGFP-ACBD3 significantly enhanced Golgi localization of the over-expressed KDELR1 and KDELR3a beyond the level observed in control HeLa cells (Fig. 2D). While the percentage of Golgi-localized KDELR2 in control HeLa cells was relatively lower compared to other isoforms, KDELR2 also showed ~3-fold reduction in its Golgi localization upon ACBD3 depletion, which was subsequently restored to that of control HeLa cells by EGFP-ACBD3 expression (Fig. 2D).

As a control, we also tested two other proteins, including GPP130 [25, 26] and CI-MPR [27–29], that had been shown to cycle between the Golgi and the endolysosomal compartments to see if they are influenced by ACBD3 depletion. We knocked out ACBD3 in HeLa cells using CRISPR technique, as described in the methods and stained the control HeLa cells and the ACBD3 KO cells with anti-GPP130 and anti-CI-MPR antibodies, respectively. The results showed that neither GPP130 nor CI-MPR showed significant changes in their steady state localization to the Golgi in ACBD3-depleted cells (Additional file 2: Figure S2E-F), suggesting that KDELR re-distribution to the ER upon ACBD3 depletion is likely to be specific among the proteins that cycle through the Golgi complex. In addition, we found that ACBD3 depletion had no significant influence on the localization of a Golgi resident glycosyltransferase ManII and an ER chaperone Erp29, further reinforcing the observation that overall trafficking between the ER and the Golgi is not altered in ACBD3 KO cells (Additional file 2: Figure S2G-H).

### ACBD3 depletion causes re-distribution of endogenous KDELR, tagged with C-terminal 3x-FLAG-mCherry via CRISPR-Cas9 technique

Although our results showed so far that depletion of ACBD3 results in re-localization of over-expressed KDELR1-mCherry, we could not test whether this holds true for endogenous KDELR, due to lack of a good antibody. To rule out the possibility of an over-expression artifact and study the trafficking/localization of endogenous KDELR, we tagged endogenous KDELR1 gene in HeLa cells using CRISPR/Cas9 gene editing with 3xFLAG tag, followed by mCherry at its C-terminus (KDELR1^ENDO^-3xFLAG-mCherry), as illustrated in Fig. 2E and the methods. This endogenously tagged KDELR1 showed a similar peri-nuclear localization to the Golgi under confocal microscope, as observed for exogenously transfected KDELR1-mCherry (Fig. 2F; control siRNA).

Depletion of ACBD3 by RNA interference in these cells indeed resulted in similar re-distribution of endogenously tagged KDELR1 to the ER, which was subsequently restored by re-expression of RNAi-resistant EGFP-ACBD3. C-terminal 3xFLAG-mCherry tagging did not seem to interfere with the endogenous KDEL receptor trafficking and ligand binding, as (i) KDELR1^endo^-3xFLAG-mCherry successfully pulled down ACBD3 and ArfGAP1/3, like exogenously expressed KDELR1-mCherry (Fig. 2G); (ii) hGH-EGFP-KDEL expression (but not the control hGH-EGFP) resulted in re-distribution of KDELR1^endo^-3xFLAG-mCherry to the ER, suggesting that the endogenously tagged KDELR1 is likely to be functioning properly (Fig. 2H).

### Super-resolution structured illumination microscopy indicates that ACBD3 co-localizes well with endogenously tagged KDELR1

**Fig. 3:**
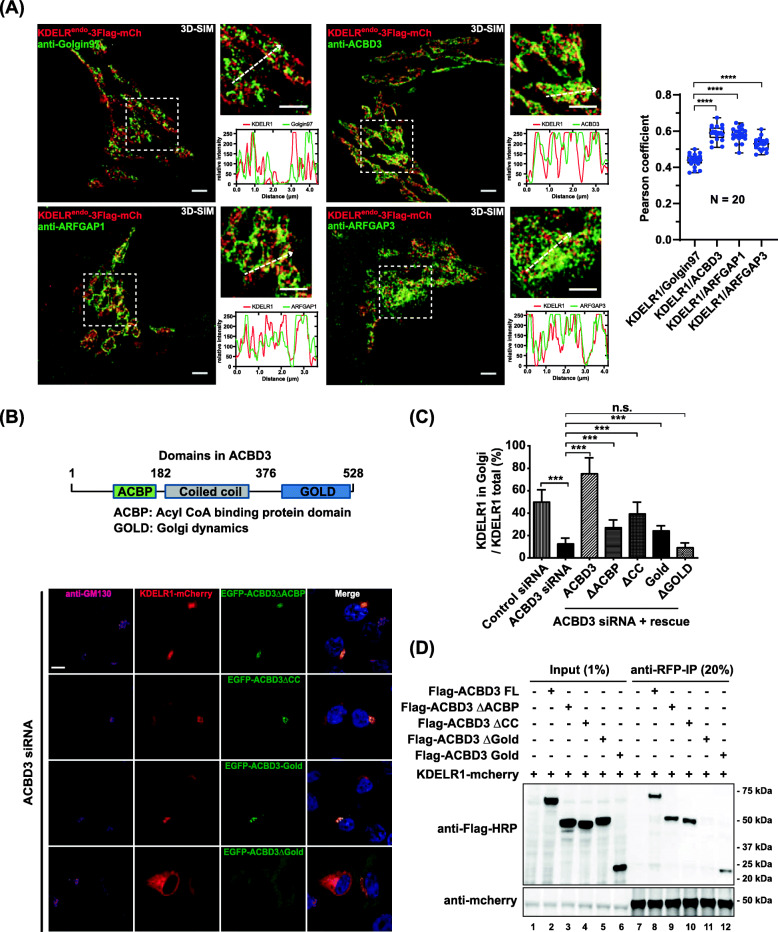
ACBD3 is required for Golgi distribution of the three isoforms of KDELR at the Golgi. **A** Super-resolution 3D-SIM images showing a high degree of co-localization between endogenously tagged KDELR1-3xFLAG-mCherry and ACBD3, ARFGAP1, or ARFGAP3, and low degree of co-localization between the endogenously tagged KDELR1 and Golgin97 (negative control). Line profiles through regions of interest were analyzed by Fiji. Scale bars = 2 μm. Co-localization (Pearson’s *R*) was determined and subjected to two-tailed, unpaired t tests (*n* = 20 cells/combination, mean and SD, ****, *p* < 0.0001). **B** Confocal micrographs of HeLa cells co-expressing KDELR1-mCherry with deletion mutants of EGFP-ACBD3 after siRNA-mediated ACBD3 knockdown, showing that the GOLD domain of ACBD3 is required for retention of KDELR1-mCherry at the Golgi. For this experiment, we sequentially deleted the ACBP domain, the CC domain, the GOLD domain, or both ACBP+CC domains and used these constructs in the knockdown and rescue experiments. **C** Histogram summarizing the rescue experiments using full length EGFP-ACBD3, EGFP-ACBD3ΔACBP, EGFP-ACBD3ΔCC, EGFP-ACBD3ΔGOLD, EGFP-ACBD3-GOLD (ΔACBP+CC), respectively. Note that rescue with full length ACBD3 actually led to ~25% increase in the percentage of Golgi-localized KDEL-R1-mCherry over the WT control cells. In addition, we observed that EGFP-ACBD3 expression often abolished ER-localization of over-expressed KDELR1-mCherry entirely. (***p < 0.001; n.s., not significant) **D** EGFP-tagged GOLD domain of ACBD3 co-immunoprecipitates with KDELR1-mCherry. KDELR1-mCherry was co-transfected with EGFP-ACBD3 WT, EGFP-ACBD3ΔACBP, EGFP-ACBD3ΔCC, EGFP-ACBD3ΔGOLD, EGFP-GOLD domain, respectively, followed by cell lysis and immunoprecipitation using anti-RFP agarose beads. Experiments were repeated three times and representative western blots are shown here. Scale bars = 10 μm

We then used super-resolution structured illumination microscopy (3D-SIM) to more precisely determine co-localization between endogenously tagged KDELR1 and endogenous ACBD3, β-COP, and ArfGAP1/3 at their physiological expression level. As an additional control, we used TGN-localized Golgin97 for these experiments. As expected, KDELR1^endo^-3xFlag-mCherry showed relatively poor co-localization with endogenous Golgin97 (Pearson coefficient=0.44±0.04; avg±SD), while showing significantly better co-localization with endogenous ACBD3 (Pearson coefficient=0.59±0.04) at this resolution (Fig. 3A). KDELR1^endo^-3xFlag-mCherry also showed better co-localization with endogenous ArfGAP1 (0.58±0.04) and ArfGAP3 (0.53±0.03) over endogenous Golgin97, respectively (Fig. 3A).

In contrast, ACBD3 showed lower co-localization with β-COP (0.42±0.03), compared to endogenous KDELR1 (0.51±0.05) and ArfGAP1/3 (0.52±0.05; 0.56±0.05), suggesting that ACBD3 is less likely to be a part of KDELR containing transport carriers (Additional file 3: Figure S3A). As a control, we also examined co-localization between ACBD3 and ArfGAP1/3. ACBD3 showed essentially no co-localization with Golgin97 (0.29±0.05) (Additional file 3: Figure S3B), while showing relatively poor co-localization with ArfGAP1/3(0.46±0.04; 0.46±0.04, respectively) using 3D-SIM.

Collectively, these results suggest that (i) co-localization between endogenous KDELR1 and ACBD3 is comparable to co-localization between endogenous KDELR1 and ArfGAP1/3 and (ii) ACBD3 is unlikely to be a component of COPI-coated buds or transport carriers for retrograde transport of KDELR to the ER.

### GOLD domain of ACBD3 is necessary and sufficient for Golgi localization of KDELR

ACBD3 is known to contain at least three distinct domains, including N-terminal Acyl-CoA Binding Protein (*ACBP*) domain, a coiled-coil domain (*CC*) in the middle, followed by a Golgi dynamics (*GOLD*) domain at the C-terminus. We gradually deleted each of these domains to determine the minimal domain required for the Golgi localization of KDELR1. Importantly, expression of EGFP-GOLD (ΔACBP/CC) in ACBD3-depleted cells was sufficient to restore KDELR1 localization to the Golgi, although there was a noticeable reduction in the rescue efficiency, compared to the full length ACBD3 (Fig. 3B, C).

This reduction in rescue efficiency may be attributed to less efficient targeting of EGFP-GOLD, compared to the full length (FL) ACBD3 (Fig. 3B). Consistent with these results, both FLAG-ACBD3 FL and FLAG-GOLD domain were co-immunoprecipitated with KDELR1-mCherry, whereas FLAG-ACBD3ΔGOLD was not (Fig. 3D). Collectively, these data further demonstrate that ACBD3 may play a crucial role in the trafficking and function of KDELR via its GOLD domain.

### ACBD3 depletion does not significantly influence conventional secretory pathway and ER-to-Golgi trafficking of KDELR

One hypothesis to explain KDELR re-distribution in ACBD3-depleted cells could be that ACBD3 depletion might have caused an overall inhibition in the ER-to-Golgi anterograde transport, leading to accumulation of KDELR in the ER. To explore this possibility, we examined secretion of several secretory cargo proteins, including a VSVG-tsO45-GFP, Transferrin receptor (TfR) fused to FM4, a conditional aggregation system, another synthetic cargo YFP-GL-GPI, and endogenous MMP-2, as described in the methods. Since leakage of ER-resident chaperones to the Golgi is known to coincide with anterograde transport of secretory cargo proteins, we assumed for these assays that anterograde transport of KDELR is likely to follow the conventional ER exit route and COPII-dependent pathway to the Golgi as well [30].

The results from the protein secretion assays (Additional file 4: Figure S4A-D) clearly showed that none of the secretory cargo proteins we tested showed significant changes in their secretion to the plasma membranes in ACBD3-depleted cells, compared to the control HeLa cells. Thus, altered re-distribution of KDELR to the ER in ACBD3-depleted cells is highly unlikely to result from an overall alteration of conventional protein secretory pathway.

**Fig. 4:**
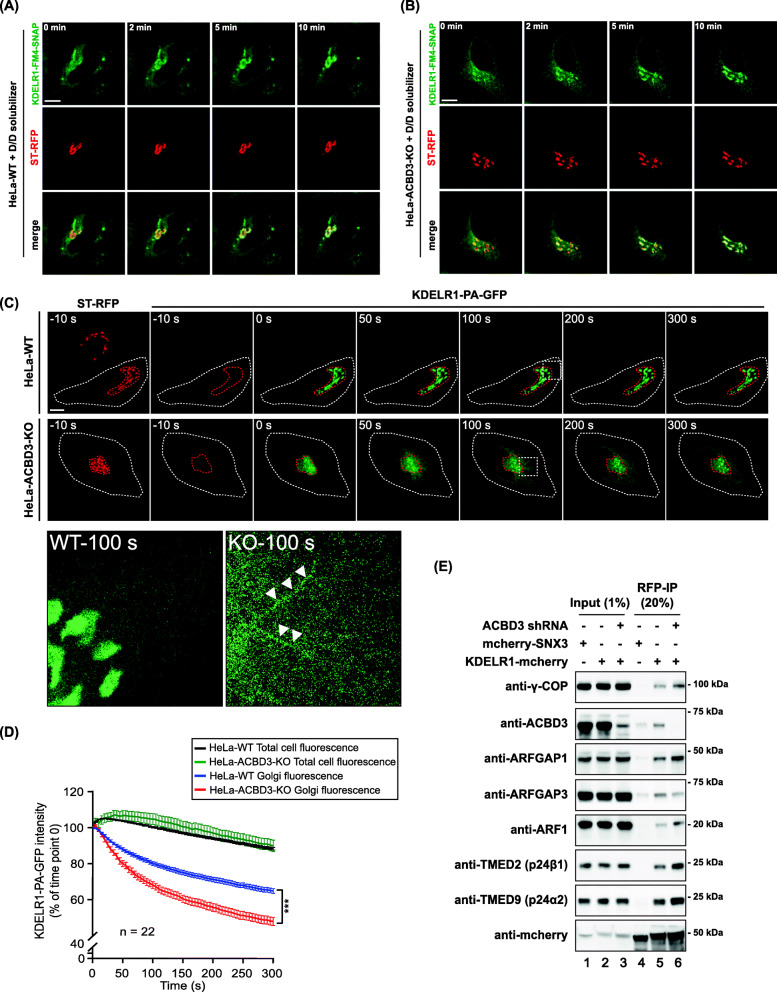
Photoactivation experiments reveals that knockout of ACBD3 results in acceleration of KDELR1 retrograde transport. **A**, **B** HeLa or HeLa-ACBD3-KO cells were co-transfected with KDELR1-FM4-SNAP and ST-RFP. After D/D solubilizer treatment, ER-Golgi antegrade transport of KDELR1-FM4-SNAP is monitored by live cell imaging acquired every 30 s for 10 min. Imaging sequences at the indicated time points are presented here. Scale bars = 10 μm. **C** To measure the amount of KDELR1 outflux from the Golgi, WT, or ACBD3-depleted HeLa cells were co-transfected with sialyltransferase-RFP (ST-RFP, a Golgi marker) and photoactivatable KDELR1-PA-GFP plasmids for 18 h. The KDELR1-PA-GFP in the Golgi were then activated by selecting an ROI of ST-RFP-positive region for intense 405-nm laser irradiation and the transport out of the Golgi are monitored by live cell imaging acquired every 5 s for 5 min. Imaging sequences prior to photoactivation (−10 s), immediately after photoactivation (0 s) and the indicated times following photoactivation are presented here. Magnified regions of interest (indicated by white boxes) from WT and ACBD3-KO cells at 100 s time point shows Golgi-derived tubules which are highlighted by white arrowheads. Scale bars = 10 μm. **D** The intensity of photoactivated GFP remaining in the Golgi area was expressed as a percentage of the intensity of photoactivated GFP at time point 0 and plotted as a function of time. The integrated fluorescence over the entire cell was normalized to the integrated fluorescence at time point 0 as a function of time (green and black lines), showing the photobleaching effect during live imaging, serving as a control for fluorescence (*n* = 22). (***p < 0.001) **E** Co-immunoprecipitation experiments indicate that ACBD3 depletion results in increased association of p24, ArfGAP1, coatomer, and Arf1 with KDELR1-mCherry.

In order to more directly test whether anterograde trafficking of KDELR from the ER to the Golgi is altered in ACBD3-depleted cells, we constructed KDELR1-FM4-SNAP to create a controlled wave of KDELR using D/D solubilizer [31]. KDELR1-FM4-SNAP was aggregated in the ER in both the control HeLa and ACBD3 KO cells. Upon addition of D/D solubilizer, KDELR1-FM4-SNAP was rapidly released from the ER and transported to the Golgi within 10 min in both the control and ACBD3 KO cells (Fig. 4A, B), demonstrating that ACBD3 depletion does not exert a significant influence on anterograde trafficking of KDELR1 from the ER to the Golgi.

### Photoactivation experiments show that retrograde trafficking KDELR1 is accelerated in ACBD3 KO cells

We then asked whether retrograde transport of KDELR might have been altered in cells lacking ACBD3, causing increased re-distribution of the receptor to the ER. To test this hypothesis, we prepared KDELR1 fused to photoactivatable GFP (PA-GFP) and studied the dynamics of retrograde transport of the receptor in both wild type (WT) HeLa cells and ACBD3-knockout cells. Sialyl transferase fused to RFP (ST-RFP) was co-transfected with KDELR1-PA-GFP to estimate the Golgi region for photoactivation. The amount of photoactivated KDELR1-PA-GFP remaining at the Golgi was monitored by fluorescence time lapse microscopy with images being collected every 5 s over a period of 5 min.

In ACBD3 knockout cells, significantly more photoactivated KDELR1-PAGFP appeared to be transported out of the Golgi, compared to the control HeLa cells. The amount of KDELR1 outflux from the Golgi was greater by approximately twofold in ACBD3 KO cells during the 300 sec period (Fig. 4C, D; Additional file 6: Video S1 and Additional file 7: Video S2). Since the initial fraction of KDELR1 at the Golgi upon ACBD3 KO is much lower than the control HeLa cells (10±8% in ACBD3 KO cells vs. 50±10 % in control cells) due to receptor re-location to the ER, it is likely that the number of photoactivated KDELR1-PAGFP would also be significantly lower in ACBD3 KO cells. Therefore, the relative KDELR outflux from the Golgi in ACBD3 KO cells over the control cells would be greater than our estimation.

Of note, we frequently observed clear tubular emanations of KDELR1-PAGFP around the perinuclear region of ACBD3 KO cells during the experiments (Fig. 4C; insets), suggesting that tubular transport carrier might be utilized for KDELR trafficking, although it does not mean that vesicular transport may also be used for KDELR trafficking. Taken together, our data suggest that accelerated retrograde trafficking of KDELR might contribute to increased re-distribution of KDELR to the ER in ACBD3-depleted cells.

In support of these results, KDELR1 co-immunoprecipitated significantly more ArfGAP1, p24, and Arf1 as well as γ-COP from ACBD3-depleted cell lysates (Fig. 4F), suggesting that KDELR1 interaction with ArfGAP1/Arf1/p24/coatomers significantly increases, resulting in more frequent KDELR trafficking upon ACBD3 depletion. Interestingly, however, Shiga Toxin-B trafficking from the Golgi to the ER was also shown to be accelerated in ACBD3 KO cells, compared to the control HeLa cells (Additional file 4: Figure S4E-F), indicating that other KDEL-independent retrograde trafficking may also be influenced by ACBD3 depletion, which will be revisited in the later part of this study.

### KDELR forms mutually exclusive protein complexes with ACBD3 and ArfGAPs

**Fig. 5:**
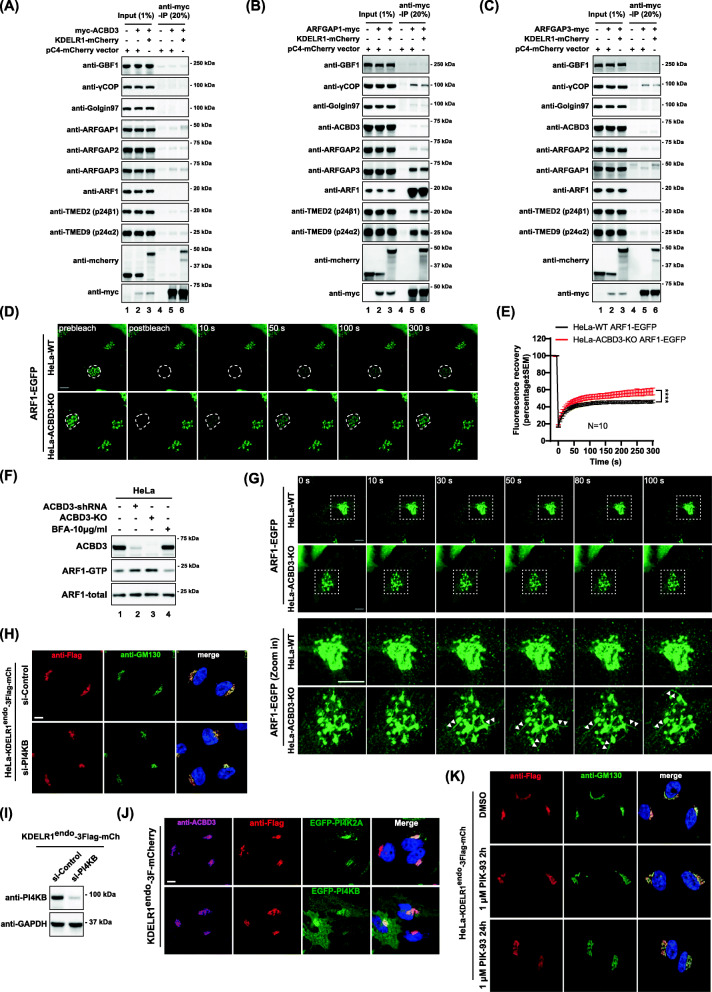
ACBD3 depletion alters KDELR association with Arf1, promoting Arf1-dependent tubular carrier formation at the Golgi. **A**–**C** myc-ACBD3, ARFGAP1-myc, or ARFGAP3-myc can efficiently pull down KDELR1-mCherry, but myc-ACBD3 does not co-immunoprecipitate with the key components of the retrograde trafficking machinery. ARFGAP1-myc co-immunoprecipitates with γCOP, ARFGAP3, ARF1, and p24 proteins, while ARFGAP3-myc only co-immunoprecipitates with γCOP. HeLa cells were co-transfected KDELR1-mCherry with myc-ACBD3, ARFGAP1-myc, or ARFGAP3-myc, respectively, and the extracts were immunoprecipitated with a myc-trap agarose beads. The lysates and the immunoprecipitates were analyzed by western blotting, as indicated. **D**, **E** Kinetics of Arf1-EGFP binding to and dissociation from Golgi membranes in WT and ACBD3-KO HeLa cells. **D** Representative FRAP experiments in WT or ACBD3-KO HeLa cells transiently expressing Arf1-EGFP. The Golgi was selectively photobleached (white circle). The first frames show an initial prebleached image. After photobleaching, image frames were selected at times 10, 50, 100, and 300 s. Scale bar, 10 μm. **E** Quantification of Golgi intensity in Arf1-EGFP–expressing WT or ACBD3-KO HeLa cells. Statistical analysis was performed using two-tailed, paired *t* test (mean±SEM, *N* = 10, ****p < 0.0001). **F** HeLa WT, ACBD3-KD (stably knockdown) or ACBD3-KO cells were analyzed for levels of Arf1-GTP, as described in the methods. **G** Live-cell imaging of transiently expressed Arf1-EGFP in HeLa WT or ACBD3-KO cells. Zoomed Golgi areas were shown in the bottom panels. Examples of Golgi-derived ARF1-rich tubules are highlighted by arrowheads. **H** Confocal images of KDELR1^endo^-3xFlag-mCherry in HeLa cells showing that depletion of PI4KB does not result in re-distribution of KDELR1^endo^-3xFlag-mCherry to the ER in vivo. Scale bars = 10 μm. **I** Western blots showing depletion of PI4KB, by RNA interference. **J** Confocal images of KDELR1^endo^-3xFlag-mCherry in HeLa cells showing that overexpression of PI4KB does not result in its re-distribution to the ER in vivo. **K** Golgi localization of KDELR1^endo^-3xFlag-mCherry is unaffected by chemical inhibition of PI4K using PIK-93. Scale bars = 10 μm

We next asked whether ACBD3 may be a part of KDELR/ArfGAPs complex in vivo, as it has been well known that ArfGAPs play important roles in retrograde trafficking of KDELR to the ER [20, 21]. Strikingly, reciprocal co-IP experiments showed that myc-ACBD3, ArfGAP1-myc, or ArfGAP3-myc pulled down KDELR1-mCherry individually, but showed no interaction with one another (Fig. 5A–C), except for ArfGAP1, which co-immunoprecipitated some ArfGAP3. As reciprocal IP experiments with ArfGAP3 (Fig. 5C) failed to show ArfGAP1 band, it is also possible that over-expression of ArfGAP1-myc may influence ArfGAP3’s role in KDELR trafficking within the Golgi. Despite of this puzzling result, these results strongly suggest that KDELR1 may form functionally distinct protein complexes with ACBD3, ArfGAP1, and ArfGAP3, respectively.

Unexpectedly, ArfGAP1 (but not ACBD3 nor ArfGAP3) also pulled down Arf1 and p24 complex (Fig. 5B), indicating that increased Arf1/p24 in KDELR immunoprecipitates upon ACBD3 depletion (Fig. 4F) is likely to be a part of ArfGAP1/KDELR complex. As p23/p24 complex had previously been shown to recruit Arf1-GDP to the Golgi membranes [32], ArfGAP1-KDELR is likely to merge into Arf1/p23/p24-enriched membrane domains for transport carrier formation in ACBD3-depleted cells.

### ACBD3 depletion alters KDELR association with Arf1 and Arf1-dependent tubular carrier formation at the Golgi in PI4P-independent manner

We then posited that increased KDELR trafficking via tubular transport carriers might indicate more dynamic membrane association of Arf1 and increased level of activated Arf1-GTP in these cells [33–36]. In order to study Arf1 membrane dynamics, FRAP experiments was performed using Arf1-EGFP to test if ACBD3 depletion results in altered Arf1 association with the Golgi membranes. The results showed that fluorescent recovery of the photobleached Arf1-EGFP at the Golgi was noticeably faster in ACBD3-depleted cells, as shown in Fig. 5D, E, suggesting that Arf1 may be in more activated state in ACBD3 KO cells.

To assess the relative level of GTP-bound Arf1 in ACBD3-depleted cells, we used GST-GGA3 VHS-GAT binding assay, as described previously by others [37, 38]. The results from this assay clearly demonstrated that Arf1-GTP level increased in both ACBD3 knockdown and knockout cells, compared to the control HeLa cells (Fig. 5F; lane 2 and 3, respectively) and Brefeldin A-treated cells (a negative control).

Increased Arf1-GTP level in ACBD3-depleted cells may be closely associated with membrane tubule formation at the Golgi [33, 36]. Thus, we examined whether ACBD3 depletion increases Arf1-GTP-dependent tubule formation for accelerated retrograde transport of KDELR1. To this end, we transfected control HeLa cells and ACBD3 KO cells with Arf1-EGFP construct and monitored Arf1-positive membrane tubular carrier formation at the Golgi by time lapse confocal microscopy, as described in the methods. Strikingly, a noticeable increase in Arf1-positive membrane tubules was readily observed in ACBD3 KO cells, compared to the control HeLa cells (Fig. 5G; Additional file 8: Video S3 for control, Additional file 9: Video S4 for ACBD3 KO cells).

PI4P is an important regulator of transport carrier formation at the Golgi [39–42] and ACBD3 is known to function as one of the docking factors for PI4KB [43–45]. Thus, we either knocked down PI4KB in HeLa cells by RNAi or over-expressed EGFP-PI4KB or treated the cells with PI4K inhibitor (PIK-93), and studied whether potentially altered PI4P level may be important for accelerated retrograde transport of KDELR to the ER in ACBD3-depleted cells. The results showed that (i) depletion of PI4KB had no effect on steady state localization of the endogenously tagged KDELR to the Golgi (Fig. 5H, I); (ii) over-expression of neither PI4KB nor PI4K2A (a control) caused re-distribution of the endogenously tagged KDELR (Fig. 5J); (iii) PIK-93 treatment up to 24 hours also did not influence KDELR localization at steady state, further confirming that PI4P plays a negligible role, if any, for altered KDELR localization in ACBD3-depleted cells (Fig. 5K).

### Both KDEL cargo expression and ACBD3 depletion similarly increase KDELR binding to PKA-Cα

**Fig. 6:**
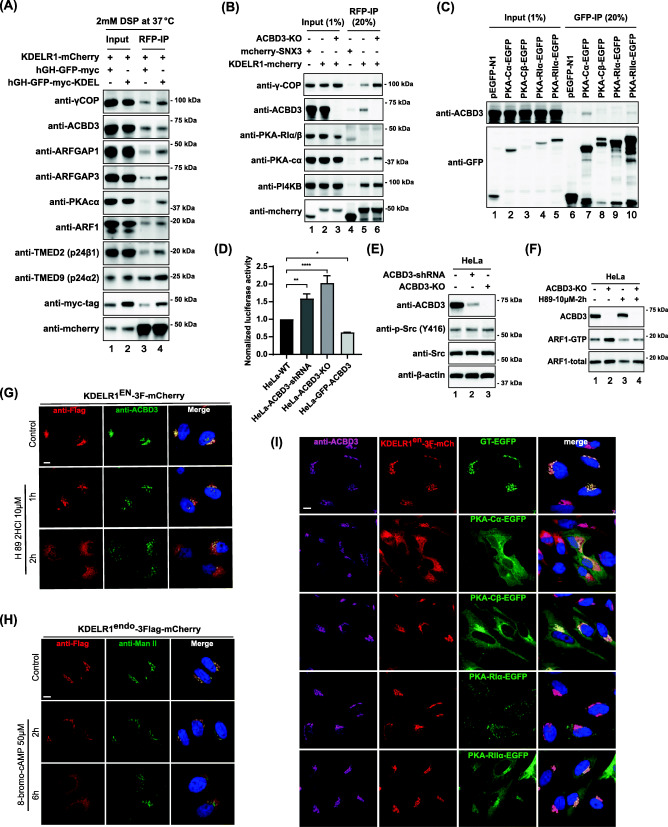
Activation of PKA and increased Arf1-GTP level plays a crucial role in KDELR relocalization. **A** Effect of the co-expression of KDEL-cargo (hGH-GFP-myc-KDEL) on KDELR1-mCherry co-immunoprecipitation in HeLa cells. HeLa cells co-transfected KDELR1-mCherry with hGH-GFP-myc or hGH-GFP-myc-KDEL were firstly treated with DSP crosslinker in PBS at 37 °C for 30 min. Cells were lysed and the extracts were immunoprecipitated with anti-RFP agarose beads. **B** Effect of ACBD3-KO on KDELR1-mCherry co-immunoprecipitation in HeLa cells. HeLa-WT or ACBD3-KO cells transfected with KDELR1-mCherry (mCherry-SNX3 transfection as a control) were lysed and immunoprecipitated with anti-RFP agarose beads. **C** Both PKA-Cα-EGFP and PKA-RII-EGFP co-immunoprecipitate with ACBD3 in HeLa cells. **D** Effect of ACBD3-KD/KO or ACBD3 over-expression on the luciferase expression in HeLa cells, expressing the CRE-controlled luciferase. HeLa-WT, ACBD3 stable knockdown, ACBD3-KO, or EGFP-ACBD3 stably over-expressing cells seeded in 24-well plate were transfected with pCRE-luciferase plasmid. Cells were then lysed, and equal protein amounts of lysate per condition were assayed using a luciferase assay system. Statistical analysis was performed using one-way ANOVA with a Tukey’s post hoc test (mean±SD, *p < 0.05; **p < 0.01; ****p < 0.0001). **E** HeLa WT, ACBD3-KD, or ACBD3-KO cells were analyzed for phosphorylation levels of Src family. **F** PKA inhibition by H89 decreases ARF1-GTP level in ACBD3-KO HeLa cells. HeLa-WT or ACBD3-KO cells were firstly treated with H89 for 2 h and then analyzed for levels of Arf1-GTP. Equal aliquots of lysates were analyzed for Arf1-GTP by using ARF1 activation assay kit utilizes pull-down with GST-GGA3. **G**, **H** Confocal images of KDELR1^endo^-3xFlag-mCherry in HeLa cells showing that PKA inhibition by H89 does not result in re-distribution of KDELR1^endo^-3xFlag-mCherry to the ER, while PKA activation by 8-Bromo-cAMP results in re-distribution of KDELR1^endo^-3xFlag-mCherry to the ER in vivo without affecting ManII localization. **I** Confocal images of KDELR1^endo^-3xFlag-mCherry in HeLa cells showing that only over-expression of PKA-Cα-EGFP results in re-distribution of KDELR1^endo^-3xFlag-mCherry to the ER *in vivo*. Scale bars = 10 μm

In order to better understand mechanistic underpinning of ACBD3-mediated regulation of KDELR trafficking, we transfected HeLa cells with hGH-GFP-myc-KDEL construct for overnight that causes retrograde transport of KDELR1, as shown in Fig. 2H. Then, cells were subjected to chemical crosslinking using 2 mM DSP at 37 °C, a membrane permeable/cleavable crosslinker, to better recover all KDELR1-bound proteins, as the KDEL ligands may fall off completely due to harsh immunoprecipitation/washing steps (or pH change) and resulting KDELR conformation changes, leading to altered bound protein profile during immunoprecipitation experiments. As shown in Fig. 6A, KDEL cargo expression significantly increased Arf1, PKA-Cα, coatomer, p24, and ArfGAP1/3, all of which indicated activation of KDELR1 retrograde transport to the ER.

Unexpectedly, KDELR pulled down increased amount of both ArfGAP1 and ArfGAP3 in cells expressing hGH-GFP-myc-KDEL cargo (Fig. 6A), whereas the receptor preferentially co-immunoprecipitated ArfGAP1 over ArfGAP3 in ACBD3 depleted cells (Fig. 4C). These results may hint that ACBD3 is likely to be involved in more selective activation of ArfGAP1-dependent retrograde transport route, while KDEL cargo activates both ArfGAP1- and ArfGAP3-mediated retrograde transport pathways. Although there are some remaining questions here, these results clearly demonstrate that ACBD3 depletion alone at least partially mimics ligand-induced activation of KDELR retrograde transport pathway.

### Activation of PKA activity and subsequent increase in Arf1-GTP level in ACBD3-depleted cells play a crucial role in KDELR relocalization to the ER

PKA had been known to phosphorylate Ser209 of KDELR cytoplasmic tail, which in turn promotes the binding of ArfGAP1 to KDELR for its retrograde transport [20]. In our experiments using chemical crosslinker, we checked whether PKA may be involved in cargo-induced KDELR trafficking and found that significantly increased PKA-Cα was pulled downed with KDELR, when cells were transfected with hGH-GFP-myc-KDEL (Fig. 6A). As ACBD3 depletion appears to partly mimic ligand-induced re-location of KDELR to the ER, we hypothesized that ACBD3 depletion might similarly influence KDELR interaction with PKA-Cα.

Thus, we performed immunoprecipitation experiments using ACBD3 KO cells to check whether ACBD3 depletion alters KDELR binding to PKA-Cα. As expected, the results clearly showed that ACBD3 depletion significantly increased KDELR binding to PKA-Cα (Fig. 6B), indicating that ACBD3 may function as a negative regulator of PKA activity on KDELR.

Since ACBD3 is also pulled down by PKA-Cα-EGFP and, to a lesser extent, PKA-RIIα-EGFP (Fig. 6C; lane 7), it is likely that KDELR may form a trimeric complex with ACBD3 and PKA-Cα at steady state. To quantitatively measure how ACBD3 depletion might influence PKA activity in vivo, we used a cAMP responsive element (CRE) controlled luciferase reporter gene assay that measures cAMP-induced PKA activity [46]. The results showed that there was a drastic increase of PKA activity (up to 2-fold increase over the control HeLa cells) in both ACBD3 KD and KO cells (Fig. 6D), demonstrating that ACBD3 depletion has not only resulted in increased binding of KDELR to PKA-Cα, but also in actual increase of PKA activity. Strikingly, we also observed that ACBD3 overexpression inhibits PKA activity to a significant extent (Fig. 6D), indicating a possible stoichiometric relationship between ACBD3 and PKA-Cα in determining overall PKA activity.

Previous studies have shown clearly that ER-Golgi trafficking of secretory cargos activates highly complex signaling cascades in KDELR-dependent manner, involving two distinct signaling pathways, Gαs-PKA and Gαq-Src [7, 47]. Therefore, in order to rule out the possibility that ACBD3 may influence the other arm of this complex signaling system of the early secretory pathway, we studied activation state of Src kinase using anti-phosphor-Src family (Y416) antibody in ACBD3-depleted cells. As shown in Fig. 6E, there were no significant changes in phosphorylation of Src family kinases in either ACBD3 KD or KO HeLa cells, suggesting that ACBD3 specifically targets and regulates Gαs-PKA signaling pathway.

Increased PKA activity in ACBD3-depleted cells seems to have stimulatory effect on Arf1-GTP level, as H89, an inhibitor of PKA, treatment suppressed increased Arf1-GTP level in ACBD3 KO cells (Fig. 6F) [48]. H89 treatment itself appeared to be disruptive to Golgi ribbon structure in HeLa cells (Fig. 6G), making it harder to analyze its effect on KDELR trafficking. To circumvent this difficulty, we chose to use 8-bromo-cAMP, a known PKA activator [47], to test whether drug-induced activation of PKA leads to KDELR trafficking to the ER. The results clearly demonstrated that drug-induced activation of PKA does result in relocalization of the endogenously tagged KDELR to the ER without affecting Golgi localization of Mannosidase II, a Golgi resident glycosyltransferase (Fig. 6H).

Combined with earlier observation on Shiga Toxin-B retrograde trafficking between the Golgi and the ER (Additional file 4: Figure S4E-F), it is possible that ACBD3-PKA may influence a broader range of retrograde pathways between the Golgi and the ER than we initially anticipated. Alternatively, this result may be caused by Shiga Toxin-B, leaking out along with KDELR retrograde transport carriers, due to its abundance at the *cis*-Golgi.

Finally, since increased binding of KDELR to PKA-Cα was observed in both KDEL-cargo expressing and ACBD3-depleted cells, we then asked whether forced over-expression of various PKA subunits can lead to KDELR relocation to the ER. To this end, HeLa cells with endogenously 3Flag-mCherry-tagged KDELR were transfected with either GT-GFP or the indicated catalytic/regulatory PKA subunits-fused to EGFP overnight and observed under confocal microscope. The results showed that only PKA-Cα over-expression caused KDELR re-localization to the ER at steady state, as shown in Fig. 6I, further suggesting that ACBD3 interaction with this catalytic subunit may play a regulatory role in KDELR trafficking.

### ACBD3 over-expression inhibits PKA activity and delays both drug-induced and cargo-induced KDELR relocalization to the ER

**Fig. 7:**
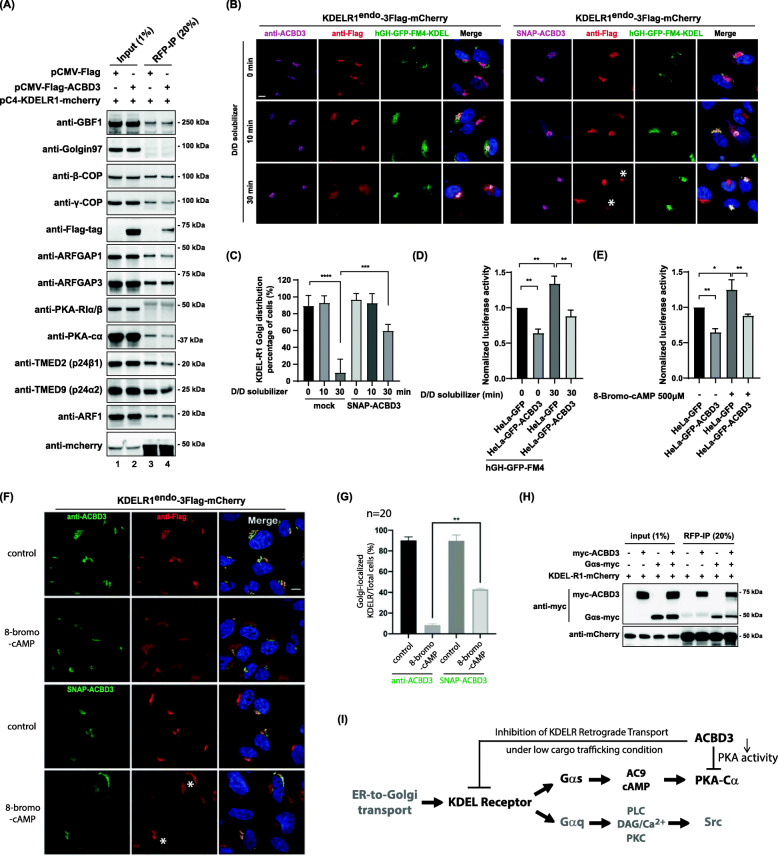
ACBD3 functions as a negative regulator of PKA activity on KDELR. **A** Effect of ACBD3 overexpression on KDELR1-mCherry co-immunoprecipitation in HeLa cells, as shown here by immunprecipitation experiments. **B**, **C** ACBD3 overexpression inhibits hGH-GFP-FM4-KDEL stimulated KDELR retrograde traffic. KDELR1^endo^-3xFlag-mCherry cells transfected with hGH-GFP-FM4-KDEL alone or co-transfected with hGH-GFP-FM4-KDEL and SNAP-ACBD3 were subjected for D/D solubilizer treatment to induce ER to Golgi trafficking of hGH-GFP-FM4-KDEL. KDELR1^endo^-3xFlag-mCherry localization was analyzed at 10min or 30min after drug treatment by confocal imaging. Scale bars = 10 μm. Histogram summarizing the percentage of cells with Golgi localized KDELR1 after D/D solubilizer treatment at indicated time point. *N*=30–40 cells. Statistical analysis was performed using two-tailed, unpaired *t* test (mean±SD, ****p < 0.0001; ***p < 0.001). **D** ACBD3 overexpression inhibits the effect of ER-to-Golgi traffic wave on PKA activity. ER-to-Golgi traffic wave was induced and PKA activity was analyzed by CRE-luciferase assay. Statistical analysis was performed using two-way ANOVA with a Tukey’s post hoc test for multiple comparisons (mean±SD, **p < 0.01). **E** ACBD3 over-expression inhibits 8-Bromo-cAMP activated PKA. Cells were treated by 500μM 8-Bromo-cAMP for 6h and then PKA activity was analyzed by CRE-luciferase assay. Statistical analysis was performed using two-way ANOVA with a Tukey’s post-hoc test for multiple comparisons (mean±SD, *p < 0.05; **p < 0.01). **F** ACBD3 over-expression inhibits 8-Bromo-cAMP stimulated KDELR1 re-distribution to the ER. KDELR1^endo^-3xFlag-mCherry HeLa cells with or without SNAP-ACBD3 expression. **G** Histogram summarizing the percentage of cells with Golgi localized KDELR1 after 8-Bromo-cAMP treatment shown in **F**. Statistical analysis was performed using two-tailed, unpaired *t* test (mean±SD, **p < 0.01). **H** Effect of ACBD3 over-expression on KDELR1-mCherry interacting with Gαs in HeLa cells, as shown here by immunoprecipitation experiments. **I** Schematic diagram depicting the proposed role of ACBD3 in regulating PKA activity and KDELR trafficking. ACBD3 likely plays a pivotal role in Golgi localization of KDELR under low cargo trafficking condition by inhibiting basal PKA activity on KDELR

As shown in Fig. 6D, we found that ACBD3 over-expression significantly inhibits PKA activity in luciferase reporter assays. To elaborate on this finding, we studied how ACBD3 over-expression may influence interaction between KDELR and its various interacting partners by immunoprecipitation experiments. To this end, we transfected KDELR-mCherry into HeLa cells with or without FLAG-tagged ACBD3, followed by immunoprecipitation using anti-RFP antibody. The results showed that there was significant reduction in the amount of ArfGAP1, PKA-Cα, γ-COP, and p24 that are pulled down with KDELR-mCherry for cells transfected with FLAG-ACBD3, compared to FLAG-vector transfected control cells (Fig. 7A). These findings were further verified in confocal experiments, in which hGH-GFP-FM4-KDEL was used as a cargo wave to induce ER relocalization of endogenously tagged KDELR in either control cells or cells over-expressing SNAP-tagged ACBD3. Consistent with the immunoprecipitation results, there was a significant delay of cargo-induced re-localization of endogenously tagged KDELR to the ER in ACBD3 over-expressing cells, compared to the control cells (Fig. 7B, C).

This delay was reflected in luciferase reporter assays for PKA activity. Although release of hGH-GFP-FM4 to the Golgi increased PKA activity in both the control and ACBD3 over-expressing HeLa cells, PKA activity was reduced by almost ~40% in GFP-ACBD3 transfected cells at 30 min time-point after cargo release to the Golgi (Fig. 7D).

Furthermore, ACBD3 over-expression similarly decreased PKA activity induced by 8-bromo-cAMP treatment, as measured by luciferase reporter assays (Fig. 7E), strongly indicating that ACBD3-dependent regulation of PKA activity likely occurs at the downstream of Gαs and cAMP generation by AC9 [47]. This observation was corroborated by confocal experiments, in which ACBD3 over-expression caused a significant delay in 8-bromo-cAMP-induced KDELR relocalization to the ER, compared to the control cells (Fig. 7F, G).

We observed that, while ACBD3 over-expression does not completely inhibit KDELR relocalization to the ER by 8-bromo-cAMP treatment, there was a clear decrease in the KDELR retrograde transport to the ER in cells transfected with SNAP-ACBD3. Taken together, these results demonstrated that ACBD3 is a novel regulator of KDELR-PKA interaction and PKA activity, functioning at the downstream of Gαs-AC9/cAMP at the Golgi.

### ACBD3 over-expression does not alter KDELR interaction with Gs

Lastly, in order to confirm that ACBD3-dependent regulation of KDELR trafficking occurs at the level of PKA, rather than via more upstream Gαs, we investigated whether ACBD3 over-expression alters KDELR interaction with Gαs by co-transfecting Gαs-myc and KDELR1-mCherry with or without myc-ACBD3, followed by cell lysis and immunoprecipitation using anti-RFP-antibody. The results showed that ACBD3 over-expression does not significantly influence KDELR interaction with Gαs (Fig. 7H), validating our earlier results obtained with 8-bromo-cAMP (Fig. 7E–G).

## Discussion

Our new study shows that ACBD3 is a novel binding partner of KDELR, which drastically influences KDELR trafficking and retention at the Golgi by regulating PKA-Cα-dependent activation of KDELR. Based on our data, ACBD3-dependent inhibition of PKA activity on KDELR is likely to result in suppression of ArfGAPs/Arf1/p24-mediated tubulovesicular carrier formation under low cargo trafficking condition, facilitating Golgi localization of KDELR at steady state. Because ACBD3 binding to KDELR in KDEL cargo expressing cells did not show any meaningful change (Fig. 6A), we prefer the model where ACBD3 plays a more regulatory role for PKA activity on KDELR, rather than functions as a physical lynchpin for KDELR retention at the Golgi, although we cannot completely exclude this possibility yet.

Overall, ACBD3’s role seems to inhibit PKA activity on nearby KDELR pool, thus effectively blocking their retrograde trafficking at basal PKA activity level. In this way, ACBD3 may contribute to Golgi localization of KDELR by suppressing KDEL-ligand-independent PKA activity at steady state. The fact that ACBD3 does not significantly influence either KDELR-Gαs interaction or activation state of Src family kinase reinforces the notion where ACBD3 specifically targets and regulates PKA activity in KDELR-dependent manner.

Our new results using 8-bromo-cAMP as an inducer of KDELR retrograde trafficking to the ER are highly consistent with the previous studies by Luini and his colleagues, as his group had shown that depletion of AC9 inhibits KDELR trafficking, whereas depletion of PDE7A1 stimulated the receptor trafficking to the ER [47].

On the other hand, ArfGAPs have long been known to be involved in COPI-dependent retrograde transport carrier formation at the Golgi [34, 49, 50]. Our results showed that ACBD3 depletion leads to increased ArfGAP1/Arf1 binding to KDELR, whereas KDEL-cargo increased binding of both ArfGAP1/3 and Arf1 to KDELR (compare Figs. 4C and 6A). These findings suggest that ACBD3 depletion does not fully mimic KDEL-cargo-induced activation of KDELR trafficking.

Nonetheless, both super-resolution imaging of ACBD3 and ArfGAP1/3 by 3D-SIM and the reciprocal co-immunoprecipitation results indicate that these three proteins are highly likely to form mutually exclusive complexes with KDELR at steady state. ArfGAP3, but not ArfGAP1, had been shown previously to require coatomers for membrane recruitment at the Golgi [49, 50]. In addition, ArfGAP3 has been shown to function for CI-MPR trafficking between the TGN and the endosomal compartments [51]. Therefore, it is possible that ArfGAP3 may be involved in post-Golgi trafficking of KDELR at the trans-Golgi network [12, 51].

We cannot determine precisely what fraction of KDELR would be subjected to retrograde transport via tubular carriers vs. COPI-vesicles at the moment, based on our study. As ArfGAP1 seems more tightly associated with Arf1-GTP at the Golgi, compared to ArfGAP3 (Fig. 5B), it may play more important role in tubular carrier formation for KDELR transport, although this hypothesis needs to be further investigated in a future study.

Based on these findings, we propose a following model (Fig. 7I), in which ACBD3 keeps a pool of KDEL-cargo free KDELR from retrograde trafficking pathway (presumably activatable at basal activity of PKA-Cα in the absence of ACBD3). This inhibitory activity by ACBD3 likely plays a pivotal role in Golgi localization of KDELR at steady state. Once KDEL-cargo binds the receptor, the retrograde pathway is likely to be further activated through both Gαs-PKA and Gαq-Src signaling cascades [7, 47], leading to PKA-mediated phosphorylation of KDELR cytoplasmic tail and ArfGAP1/Arf1-dependent transport carrier formation.

In ACBD3-depleted cells, the ACBD3-dependent restriction is removed and bypassed by constitutively activated PKA activity on KDELR, leading to leakage of KDELR to its retrograde pathway. In contrast, excess ACBD3 may compete with PKA-Cα for its binding to KDELR in ACBD3-overexpressing cells (Fig. 7A), further reducing PKA-Cα activity and delays KDEL-cargo-induced retrograde trafficking of KDELR. Therefore, these results suggest that there may be an inverse stoichiometric relationship between ABCD3 expression level and PKA activity at the Golgi.

Lastly, it is not unexpected that ACBD3 function as a regulator of PKA activity for KDEL receptor trafficking, as it was originally known as PAP7, a PBR/PKA-interacting protein, at the Golgi and the mitochondria [52–54]. Thus, our new study reveals for the first time that (i) KDELR localization to the Golgi at steady state is dependent on an active retention mechanism, as opposed to the widely accepted notion that KDELR localization to the Golgi results from a dynamic balance between antergrade and retrograde trafficking of the receptor between the ER and the Golgi, and (ii) ACBD3-dependent regulation of KDELR/PKA-Cα interaction and PKA activity reveals a previously unrecognized layer of the complex control system in the early secretory pathway for maintenance of cellular homeostasis.

## Conclusions

The current study reports that there exists an elaborate retention mechanism for Golgi localization of KDEL receptor. We show that this involves ACBD3 and Protein Kinase A, both of which had been known to have diverse roles in regulating Golgi structure and function. By depleting or over-expressing ACBD3, we demonstrated that PKA activity at the Golgi can be modulated to a large extent, leading to a significant alteration in retrograde trafficking of KDELR to the ER. ACBD3 expression level not only influences PKA activity on KDELR, but it also affected physical association between KDELR and PKA-Cα, a catalytic subunit of PKA. As a whole, our study suggests that the tripartite interaction among KDELR, ACBD3, and PKA represent a previously unrecognized layer of the complex mechanism for homeostasis of the early secretory pathway.

## Methods

### Cell culture and transfection

HeLa (ATCC, CCL-2) and HT-1080 (Stem Cell Bank, Chinese Academy of Sciences) were grown in Delbecco’s modified Eagle medium (DMEM, ThermoFisher) supplemented with 10% fetal bovine serum (FBS, ThermoFisher). Transfection of DNA constructs and siRNAs was performed using Lipofectamine 2000 and RNAiMAX (ThermoFisher), respectively, according to the manufacturer’s instructions. For DNA expression, cells were transfected 24 h before Co-IP experiments and 18 h for immunofluorescence (IF) experiments. For siRNA knockdown, cells were transfected 72 h before experiments. HeLa cells were authenticated by STR profiling. The authentication of HT-1080 is provided by Stem Cell Bank, Chinese Academy of Sciences. All cell lines were routinely tested for the mycoplasma contamination and were negative.

### Antibodies, reagents, siRNAs, and shRNA

Following antibodies were used in this study: anti-Giantin (ab174655, Abcam), anti-Golgin160 (ab96080, Abcam), anti-GRASP55 (10598-1-AP, Proteintech), anti-Syntaxin 5 (110053, Synaptic Systems), anti-ACBD3 (HPA015594, Sigma-Aldrich), anti-ACBD3 (H00064746-B01P, Abnova), anti-GM130 (610822, BD bioscience), anti-Golgin97 (#13192, Cell Signaling Technology), anti-β-COP (ab2899, Abcam), anti-γ-COP (sc-393615, Santa cruz), anti-ARFGAP1 (ab204405, Abcam), anti-ARFGAP2 (ab133768, Abcam), anti-ARFGAP3 (15293-1-AP, Proteintech), anti-Myc-tag (2278S, CST), Streptavidin-HRP (#3999, Cell Signaling Technology), anti-mCherry (ab167453, Abcam), anti-SNAP tag (P9310S, NEB), anti-GFP (11814460001, Roche), anti-GFP (ab6556, Abcam), anti-Flag (F1804, Sigma-Aldrich), anti-Flag-HRP (2044s, Cell Signaling Technology), anti-GBF1 (ab86071, Abcam), anti-ARF1 (10790-1-AP, Proteintech), anti-TMED2 (11981-1-AP, Proteintech), anti-TMED9 (21620-1-AP, Proteintech), anti-PI4KB (611816, BD bioscience), anti-PKA-cα (#4782, Cell Signaling Technology), anti-PKA-RIα/β (#3927, Cell Signaling Technology), and anti-GAPDH (KC-5G5, Kangchen Bio-tech). Anti-Rabbit Alexa Fluor 488 (A21441), Alexa Fluor 568 (A10042), Alexa Fluor 647 (A21245), anti-Mouse Alexa Fluor 488 (A21200), Alexa Fluor 568 (A10037), and Alexa Fluor 647 (A21236) for IF were obtained from ThermoFisher.

All common reagents were purchased from Sigma-Aldrich, unless otherwise mentioned. Brefeldin A, PIK-93, 8-Bromo-cAMP, and H89 were purchased from Selleck.

The sequence of the non-targeting control siRNA was UUCUCCGAACGUGUCACGU. The siRNAs targeting the 3′-UTR of ACBD3 and siRNAs targeting Giantin, GRASP55, Golgin160, ARFGAP1, ARFGAP2, and ARFGAP3 were custom designed by Shanghai GenePharma, China. The sequences are as follows: ACBD3 siRNA-1: GCAUUAGAGUCACAGUUUA, ACBD3 siRNA-2: GCUGAAGUUACAUGAGCUA; Giantin siRNA-1: GCUAAAGAGUGUAUGGAAA, Giantin siRNA-2: GCUGCUGCAGAGAAUAAUA; GRASP55 siRNA-1: GGCAUUGGAUAUGGUUAUU, GRASP55 siRNA-2: CCAGCUGUCCUCAGUUAAU; Golgin160 siRNA-1: GCACCUGAAACUCGAGAAU, Golgin160 siRNA-2: GGAAGAAGGUACCGAGGAA; ARFGAP1 siRNA-1: AAGGUGGUCGCUCUGGCCGAAG, ARFGAP1 siRNA-2: GCAACAUAGACCAGAGCUU; ARFGAP2 siRNA-1: AGCAGGAAGUGUAUCUCUG, ARFGAP2 siRNA-2: GAGCUCCAGAUUGAUCGUA; ARFGAP3 siRNA-1: GGUUUCAGUUGCGAUGCAU, ARFGAP3 siRNA-2: GCAAUAGCAGAACCAUCUU. We combined siRNA-1 and siRNA-2 to get high knockdown efficiency for the experiments.

The stable knockdown of ACBD3 was achieved by infecting target cells using lentivirus expression of ACBD3-shRNA (GCTGAAGTTACATGAGCTACA). The lentivirus was packaged and commercially provided by Shanghai GenePharma, China. Cells were infected with the lentivirus expressing ACBD3-shRNA using Polybrene (Sigma) overnight. Two days after infection, the cells were cultured in puromycin (0.3–1 μg/ml, ThermoFisher) for 2 weeks.

### BioID

For KDELR1-BioID, HeLa cells (four 10-cm dishes of cells for each experimental condition) were transfected with pcDNA3.1-KDELR1-Myc-BirA*. In a control experiment, Hela cells were transfected with pcDNA3.1-KDELR1-Myc. After 24-h transfection, cells were incubated for 6 h in complete media supplemented with 50 μM biotin. After three times PBS washes, cells were lysed at 25°C in 1 mL lysis buffer (50 mM Tris, pH 7.4, 500 mM NaCl, 0.4% SDS, 2% Triton X-100, 5 mM EDTA, 1 mM DTT, and 1× Complete protease inhibitor (Roche)) and sonicated to disrupt any visible aggregates. After sonication, an equal volume of 4°C 50 mM Tris (pH 7.4) was added before additional sonication and then centrifuged at 16,000×*g* for 20 min at 4°C. Supernatants were incubated with 400 μl Streptavidin Agarose (S1638, Sigma Aldrich) for 1.5 h at room temperature. Beads were collected and washed three times for 5 min at 25°C in 1 mL 1 x lysis buffer (50 mM Tris, pH 7.4, 250 mM NaCl, 0.2% SDS, 1% Triton X-100, 2.5 mM EDTA, 0.5 mM DTT, and 1 x Complete protease inhibitor (Roche)). This was repeated three times with PBS. The bound proteins were removed from the agarose beads by boiling in 200 μL of Laemmli SDS-sample buffer at 95°C for 15 min. Twenty-five percent of the sample was reserved for Western blot analysis, and the remaining beads (75%) were reserved for analysis by mass spectrometry.

### Confocal microscopy

Cells grown on glass coverslips in 24-well plates were fixed for 10 min with 4% paraformaldehyde (PFA), permeabilized in permeabilization Buffer (0.3% Igepal CA-630, 0.05% Triton-X 100, 0.1% IgG-free BSA in PBS) for 3 min, and blocked in blocking buffer (0.05% Igepal CA-630, 0.05% Triton-X 100, 5% normal goat serum in PBS) for 60 min. Primary and secondary antibodies were applied in blocking buffer for 1 h. The nucleus was stained with Hoechst-33342 (sc-200908, Santa cruz Biotechnology). Cells were washed three times with wash buffer (0.05% Igepal CA-630, 0.05% Triton-X 100, 0.2% IgG-free BSA in PBS) and twice with PBS. Coverslips were mounted using ProLong Gold Antifade Reagent (ThermoFisher). Dip coverslip in diH2O before mounting to prevent salt contamination. Images were acquired with a Zeiss LSM880 confocal microscope using a 63× Apochromat oil-immersion objective. 3D-structured illumination microscopy (SIM) imaging was acquired using Nikon N-SIM microscope.

### Split YFP assay

The split YFP experiment was performed to confirm the interaction between KDELR1 and ACBD3 in living cells. Human KDELR1 cDNA was inserted upstream of the C-terminal half of EYFP (cYFP, aa 155-238) in the pC4 vector. Human ACBD3 cDNA were fused downstream of the N-terminal half of EYFP (nYFP, aa 1-154) in the pC4 vector. HeLa cells were transfected with both plasmids for 18 h and observed by Zeiss 880 confocal microscope. For control experiments, either of these two plasmids was transfected individually.

### Live cell imaging and fluorescence recovery after photobleaching (FRAP)

For photoactivation experiments, HeLa WT and ACBD3-KO cells were seeded on a glass-bottom dish (35 mm diameter, In Vitro Scientific) coated with fibronectin (Millipore). After 18-h co-transfection with ST-RFP and KDELR1-PA-GFP, cells were imaged with a 63× objective on a Zeiss LSM 880 confocal microscope in an atmosphere of 5% CO2 at 37°C. Photoactivating KDELR1-PA-GFP in the Golgi was achieved by using a 405-nm laser. Images were acquired every 5 s for 5 min. The intensity of photoactivated GFP remaining in the Golgi was expressed as a percentage of the intensity of photoactivated GFP at time point 0 and plotted as function of time. The integrated fluorescence over the entire cell image normalized to the integrated fluorescence at time point 0 as function of time, showing the photobleaching effect of live imaging.

For live cell imaging of KDELR-FM4-SNAP or ARF1-EGFP, HeLa WT or ACBD3-KO cells were seeded on a glass-bottom dish (35-mm diameter, In Vitro Scientific) coated with fibronectin (Millipore). After 18-h transfection, cells were imaged with a 63× objective on a Zeiss LSM 880 Airyscan confocal microscope in an atmosphere of 5% CO_2_ at 37°C. For KDELR1-FM4-SNAP, images were acquired every 30 s for 10 min, while images were acquired every 2 s for 100 s for Arf1-EGFP.

For FRAP experiments, HeLa WT or ACBD3-KO were seeded on a glass-bottom dish (35-mm diameter, In Vitro Scientific) coated with fibronectin (Millipore). After 18-h transfection with ARF1-EGFP, the cells were imaged with a 63× objective on a Zeiss LSM 880 confocal microscope in an atmosphere of 5% CO2 at 37°C. The Golgi area was bleached using a single 488 laser pulse. Images were acquired every 5 s for 5 min. Fluorescence values in the bleached and an adjacent non-bleached area were measured using Fiji. Fluorescence recovery is represented as the ratio of the bleached to the adjacent areas, normalized to the pre-bleach and immediate post-bleach values. Experiments were repeated ten times, and the graph was plotted using average values from these experiments (± SEM).

### Split-ubiquitin membrane yeast two-hybrid system

To investigate the interactions between KDELR1 and ACBD3/ARFGAPs, the split-ubiquitin membrane yeast two-hybrid assay (BioGene) was performed as described previously (*36*). In our experiments, human KDELR1 cDNA was subcloned in frame and upstream of the C-terminal half of ubiquitin (Cub) and the artificial transcription factor LexA-VP16 in the pBT3-SUC bait vector. Human ACBD3, ARFGAP1, ARFGAP2, ARFGAP3, and Golgin97 cDNA were individually fused to the mutated N-terminal half of ubiquitin (NubG) in the pPR3-N prey vector. The NMY51 yeast strain co-transformed with bait/prey pair were spreaded onto selective medium lacking leucine and tryptophan (SD/-Leu/-Trp, DDO, Clontech). The physical binding of bait and prey was identified by colony selection in selective medium lacking adenine, leucine, tryptophan, and histidine (SD/-Ade/-Leu/-Trp/-His, Clontech) supplemented with X-α-Gal (QDO/X). Co-transformation of pTSU2-APP and pNubG-Fe65 or KDELR1-Cub-LexA-VP16 and pOST1-NubI were performed as positive controls.

### Co-immunoprecipitation (Co-IP) and immunoblotting

For Co-IP experiments, total lysates were prepared using lysis buffer (20mM HEPES, pH 7.4, 100mM NaCl, 2 mM DDM (N-Dodecyl-β-D-Maltoside, Anatrace) + 0.02% CHS (Cholesteryl hemisuccinate tris salt, Sigma-Aldrich) and protease inhibitors (Roche)). Subsequently, the total lysates were passed through a syringe needle (15 times) and then incubated at 4°C with end-over-end agitation for 1.5 h. For Co-IP using DSP crosslinker, HeLa cells co-transfected with KDELR1-mCherry and hGH-GFP-myc or hGH-GFP-myc-KDEL were firstly treated with DSP (dithiobis(succinimidyl propionate)) crosslinker (MedChemExpress, HY-118759) in PBS at 37 °C for 30 min. Cells were lysed using lysis buffer. Subsequently, the total lysates were incubated at 4°C with end-over-end agitation for 1.5 h and then sonicated briefly. The lysates were then cleared by centrifugation at 15,000×*g* for 20 min. The supernatants were incubated with anti-RFP agarose beads (M165-8, MBL life science) or anti-GFP agarose beads (D153-8, MBL life science) or myc-trap agarose beads (yta-20, Chromotek) for 4 h at 4°C with end-over-end agitation. The beads were washed two times with ice-cold lysis buffer and one time with PBS. Proteins were eluted by boiling in 2× SDS running buffer and subjected to SDS-PAGE for immunoblotting.

For immunoblotting, proteins were separated by SDS-PAGE (Genscript) and transferred onto nitrocellulose membranes (Amersham). Membranes were probed with specific primary antibodies and then with peroxidase-conjugated secondary antibodies (Jackson ImmunoResearch). The bands were visualized with chemiluminescence (Clarity Western ECL Substrate, Bio-Rad) and imaged by a ChemiDoc Touch imaging system (Bio-Rad). Representative blots are shown from several experiments.

### Arf activation assay

To assess the amount of activated Arf-GTP in cells, we performed pull-down assays by using the Arf1 pull-down activation assay Biochem Kit™, according to the manufacturer’s instruction (Cytoskeleton, #BK032-S).

### Luciferase reporter gene assay

To monitor the activity of the cAMP/PKA signaling pathway, a cAMP responsive element (CRE) controlled luciferase reporter gene assay was used. This reporter contains the firefly luciferase gene under the control of multimerized cAMP response element (CRE) located upstream of a minimal promoter. Cells seeded in 24-well plate were transfected with pCRE-luciferase plasmid. Cells were then lysed, and equal protein amounts of lysate per condition were assayed using a luciferase assay system (Promega, E4030), according to the manufacturer’s protocol. Experiments were repeated three times.

### MMP2 secretion assays

MMP2 secretion in HT1080-WT and HT1080-ACBD3-KO cells were assessed by Total MMP-2 Quantikine ELISA Kit (R&D Systems), according to the manufacturer’s protocols.

### Retrograde transport assay of Shiga toxin B-fragment

HeLa WT and ACBD3-KO cells were seeded on a glass-bottom 24-well plate (In Vitro Scientific) coated with fibronectin (Millipore). After 18-h transfection with ST-RFP, cells were incubated with the His-tagged Shiga toxin B subunit (Sigma, SML0655, 2.5μg/ml, final concentration) in DMEM+1%FBS for 45 min at 4 °C. Cells were rinsed and incubated with DMEM+10%FBS at 37 °C for indicated time points. Then cells were fixed and subjected for immunofluorescence staining.

### CRISPR/Cas9 gene editing

The pSpCas9(BB)-2A-GFP plasmid was purchased from Addgene (addgene 48138). Gene-specific single-guide RNA (sgRNA) sequences were designed using the online software (http://crispr.mit.edu) resource from the Zhang Laboratory and were cloned into pSpCas9(BB)-2A-GFP using the BbsI restriction enzyme sites. HT-1080 cells were transfected with ACBD3 sgRNA-containing plasmids. Isolation of clonal cell populations from the transfected cells can be performed 24 h after transfection by FACS into 96-well plate. Single clones were expanded and screened by immunoblotting, genomic sequencing, and functional assays. sgRNA oligonucleotides were as follows: ACBD3 sgRNA, forward, 5′-CACCGTCGCCACCTGGATCCGGTCG-3′, reverse, 5′-AAACCGACCGGATCCAGGTGGCGAC-3′.

CRISPR knockout of ACBD3 in HeLa cells were performed using a lentiviral expression vector lentiCRISPRv2. This plasmid was a kind gift from Dr. Jia Liu in ShanghaiTech University. ACBD3 sgRNA was subcloned into lentiCRISPRv2 using the BbsI restriction enzyme sites. To make lentivirus, the transfer plasmid was co-transfected with packaging plasmids pMD2.G and psPAX2 (gifts from Dr. Jia Liu). Briefly, 6-well plate of 80% confluent HEK293T cells were co-transfected using 2 μg of the transfer plasmid, 1 μg of pMD2.G and 1.5 μg psPAX2. After 60 h, viral supernatant was filtered through a 0.45-μm filter (Millipore) and used for infecting HeLa cells in 6-well plate immediately. After 48-h infection, the infected cells were selected using puromycin treatment for about 2 weeks. Then Isolation of clonal cell populations was performed by dilution into 96-well plate. Single clones were expanded and screened by immunoblotting, genomic sequencing, and functional assays.

### CRISPR/Cas9-mediated knockin of 3×Flag-mCherry fusion proteins at KDELR1 loci

We used the CRISPR/Cas9 system to create knockin cell lines stably expressing C-terminal 3×Flag-mCherry fusion proteins at KDELR1 locus. Design of the guide RNAs was carried out using the CRISPR Design Tool from the Zhang lab website (http://crispr.mit.edu) to minimize potential off-target effects. The KDELR1 genomic locus (Gene ID 10945) was targeted with the following guide RNA: 5′-GAGAGAGATGGAGAGGACCG-3′ located just after the stop codon in the KDELR1 coding sequence. The guide RNA was encoded in bicistronic expression plasmids pX330 (addgene plasmid #42230), as previously described (*37*). The homologous repair plasmid for genome editing was generated using these four PCR products: the pEGFP-N1 plasmid backbone, the left and right homology arms (~1,000 bp), and the reporter/selection cassette. The left homology arm fusing with 3×Flag-mCherry were synthesized (Genescript) and subcloned into pEGFP-N1 using AseI and NotI. Then the right homology arm was synthesized and subcloned after the Neor/Kanr resistance cassette to integrate the whole cassette and allow selection of positive recombinants with the drug G418/Geneticin (Thermo Fisher Scientific). The PAM site was mutagenized to avoid re-cutting by the Cas9. A glycin/serin rich linker was added (GSSGRDPGSGSG) before 3×Flag-mCherry.

The pX330 plasmid with the KDELR1 guide and the corresponding homologous recombination plasmid were co-transfected in HeLa cells using Lipofectamine 2000. G418 was added to the cells a week after transfection. After 2 weeks of selection, cells were subjected to single cell sorting into 96-well plates by dilution. Clones were genotyped via western blot and PCR. Two oligos (KDELR1-forward 5′-CATTTCGAGGGCTTCTTCGACCTCATC-3′ and KDELR1-reverse 5′-GTCACCCCTGGATGGGAAAGCTCTTCA-3′) were used to amplify the genomic region around the cut and then to genotype the single clones.

### MS sample preparation, analysis, and data analysis

Independent triplicates of control experiments and KDELR1-BioID experiments (75% of the beads) were prepared and analyzed by MS independently in a label free format. Samples were prepared by in-gel digestion as follows. Peptides were separated and analyzed on an Easy-nLC 1000 system coupled to a Q Exactive HF (both - Thermo Scientific). About 1 μg of peptides was separated in a home-made column (75 μm × 15 cm) packed with C18 AQ (5 μm, 300Å, Michrom BioResources, Auburn, CA, USA) at a flow rate of 300 nL/min. Mobile phase A (0.1% formic acid in 2% ACN) and mobile phase B (0.1% formic acid in 98% ACN) were used to establish a 60-min gradient comprised of 2 min of 5% B, 40 min of 5–26% B, 5 min of 26–30% B, 1 min of 30-35% B, 2 min of 35-90% B, and 10 min of 90% B. Peptides were then ionized by electrospray at 1.9 kV. A full MS spectrum (375–1400 m/z range) was acquired at a resolution of120,000 at m/z 200 and a maximum ion accumulation time of 20 ms. Dynamic exclusion was set to 30 s. Resolution for HCD MS/MS spectra was set to 30,000 at m/z 200. The AGC setting of MS and MS2 were set at 3E6 and 1E5, respectively. The 20 most intense ions above a 1.0E3 counts threshold were selected for fragmentation by HCD with a maximum ion accumulation time of 60 ms. Isolation width of 1.6 m/z units was used for MS2. Single and unassigned charged ions were excluded from MS/MS. For HCD, normalized collision energy was set to 25%.

The raw data were processed and searched with MaxQuant 1.5.4.1 with MS tolerance of 4.5 ppm, and MS/MS tolerance of 20 ppm. The UniProt human protein database (release 2016_07, 70630 sequences) and database for proteomics contaminants from MaxQuant were used for database searches. Reversed database searches were used to evaluate false discovery rate (FDR) of peptide and protein identifications. Two missed cleavage sites of trypsin were allowed. Carbamidomethylation (C) was set as a fixed modification, and oxidation (M), Acetyl (Protein N-term), and deamidation (NQ) were set as variable modifications. The FDR of both peptide identification and protein identification was set to be 1% [55]. The options of “Second peptides,” “Match between runs,” and “Dependent peptides” were enabled. Label-free quantification was used to quantify the difference of protein abundances between different samples [56, 57].

### Image processing and statistical analysis

Pearson coefficient and line intensity of 3D-SIM images were analyzed by Fiji software. Results are displayed as mean ± SD (standard deviation) of results from each experiment or dataset, as indicated in figure legends. All statistical tests were performed using Student’s *T* tests or ANOVA (GraphPad, Prism). Significance values are assigned in specific experiments. N (number of individual experiments) is noted in the figure legends.

## Supplementary Information

**Additional file 1: Figure S1.** (A) Characterization of HeLa cells expressing KDELR1 tagged with myc-BirA*. HeLa cells expressing KDELR1-myc-BirA* for 18 hours were grown in media containing biotin (50 μM) for 6 h and double labelled with streptavidin-488 (green) and anti-myc (red). (B) Lists of identified interaction partners in KDELR1-BioID with a number of identified peptides (all and unique) with their sequence coverage. (C) Immunoblotting of KDELR1-BioID samples confirmed biotinylation of the candidate interaction partners identified in mass spectrometry. Anti-GM130 and anti-Golgin97 blots were included as negative controls here. (D - J) Confocal micrographs of HeLa cells expressing KDELR1-mCherry, showing that depletion of Giantin, GRASP55, Golgin160 and ARFGAP1/2/3 do not result in ER re-distribution of KDELR1-mCherry. (K) Western blots showing depletion of Giantin, GRASP55, Golgin160, ACBD3 and ArfGAP1/2/3, respectively, by RNA interference. Scale bars = 10 μm.

**Additional file 2: Figure S2.** (A) Confocal micrographs of HeLa cells expressing KDELR1-mCherry showing that depletion of ACBD3 results in re-distribution of KDELR1-mCherry from the Golgi to the ER *in vivo* (Calnexin; ER marker). Expression of RNAi-resistant form of EGFP-ACBD3 restores perinuclear localization of KDEL receptor in the ACBD3 Knockdown cells. (B-C) ACBD3 knock-out by CRISPR/Cas9 technique in HT1080 cells result in re-distribution of KDELR1-mCherry to the ER. Confocal micrographs of WT and ACBD3-knockout HT1080 cells expressing KDELR1-mCherry showing that knockout of ACBD3 results in relocating KDELR1-mCherry from the Golgi to the ER *in vivo*. Calnexin was used as an ER-marker in these experiments. (D) Confocal results showing that hGH-GFP-KDEL expression induces exogenously expressed KDELR1-mCherry to re-distribute KDEL receptor to the ER (calnexin-positive compartment). The bar graph shows the summary of the confocal experiments. (***, *p* < 0.001) (E-F) ACBD3 depletion does not influence Golgi localization of other cycling proteins, such as GPP130 and CI-MPR. (G-H) ACBD3 depletion does not influence Golgi localization of a Golgi resident glycosyltransferase ManII nor secretion of ER-resident chaperone ERP29. scale bar = 10 μm.

**Additional file 3: Figure S3.** (A) 3D-SIM images showing that β-COP co-localized most extensively with ArfGAP3 and endogenously tagged KDELR1, followed by ArfGAP1. ACBD3 and β-COP didn’t show a significant overlap. Line profiles through regions of interest were analyzed by Fiji. Scale bars = 2 μm. Co-localization (Pearson’s R) was determined and subjected to two-tailed, unpaired t tests (*n* = 20 cells/combination, mean and SD, ****, *p* < 0.0001). (B) 3D-SIM images showing moderate co-localization between endogenous ACBD3 and endogenous ARFGAP1/3. No co-localization between endogenous ACBD3 and Golgin97, which serves as a negative control. Line profiles through regions of interest were analyzed by Fiji. Scale bars = 2 μm. Co-localization (Pearson’s R) was determined and subjected to two-tailed, unpaired t tests (*n* = 20 cells/combination, mean and SD, ****, *p* < 0.0001).

**Additional file 4: Figure S4.** Anterograde transport of secretory cargo proteins is not significantly altered in ACBD3-depleted cells. In order to investigate whether ACBD3 depletion might have affected anterograde transport between the ER and the Golgi, secretion of three different cargo proteins was tested, including TfR-RM4-SNAP (A), VSVG-tsO45-GFP (B), endogenous MMP-2 (C) and YFP-GL-GPI (D). (A-B) Briefly, plasmids encoding the indicated constructs were transiently transfected into control cells or ACBD3-depleted HeLa cells for 18 hours. Cells were then treated with cycloheximide for 2 hours, prior to induction of synchronized protein secretion by shifting temperature from 40.5 to 32 °C. (VSVG-tsO45-GFP) or treatment with D/D solubilizer drug (TfR-FM4-SNAP) for the indicated times. At the indicated timepoints, the cells were placed on ice and subjected to surface biotinylation using sulfo-NHS-LC-biotin for 30 min. The cells were then lysed, subjected to pulldown with streptavidin-agarose and analyzed by western blot. (C) For MMP2 measurement, the conditioned media from control HT1080 or ACBD3-KO HT1080 cells were collected after 18 hours incubation and added to Total MMP2 Quantikine ELISA kit for quantification, as described in the methods. (D) After 18 hrs transfection of YFP-GL-GPI, HeLa WT and ACBD3-KO cells were stained for indicated antibodies and then examined by confocal microscopy. Line profiles through regions of interest were analyzed by Fiji. (Scale bars = 10 μm) (E-F) HeLa-WT or HeLa-ACBD3-KO cells were transfected with sialyltransferase-RFP (ST-RFP, a Golgi marker) and His-tagged Shiga toxin B fragment (2.5 mg/ml final concentration in DMEM+1%FBS) was added to cells for 45min at 4°C. After the withdrawal of unbound toxin by washing for three times in ice-cold PBS, cells were incubated with DMEM+10%FBS at 37°C for indicated time points. Then cells were stained using anti-His-tag and anti-calnexin (as an ER marker) antibodies. The results show that plasma membrane-to-Golgi transport of His-tagged Shiga toxin B fragment is not altered in ACBD3-KO cells, while the Golgi-to-ER transport of His-tagged Shiga toxin B fragment is accelerated in ACBD3-KO cells. Scale bars = 10 μm.

**Additional file 5.** Raw-data-Western blotting.

**Additional file 6: Video S1.**

**Additional file 7: Video S2.**

**Additional file 8: Video S3.**

**Additional file 9: Video S4.**

**Additional file 10.** KDELR-BioID Mass Spectrometry Data.

## Data Availability

All data generated or analyzed during this study are included in this published article and its supplementary information files.

## Abbreviations

ER: Endoplasmic reticulum
GPCR: G-protein coupled receptor
KDELR: KDEL receptor
PKA: Protein Kinase A
PM: Plasma membrane
Co-IP: Co-immunoprecipitation
MYTH: Membrane yeast two-hybrid assays
SIM: Structured illumination microscopy
ACBP: Acyl-CoA Binding Protein
TfR: Transferrin receptor
PA-GFP: Photoactivatable GFP
ST: Sialyl transferase
DDM: N-Dodecyl-β-D-maltoside
CHS: Cholesteryl hemisuccinate tris salt
DSP: Dithiobis(succinimidyl propionate)
CRE: cAMP responsive element
