# Supplementary material for: ACBD3 modulates KDEL receptor interaction with PKA for its trafficking via tubulovesicular carrier
Source: BMC Biol. 2021 Sep 7;19:194. doi: 10.1186/s12915-021-01137-7 (PMC8424950; doi:10.1186/s12915-021-01137-7)

Additional File 1: Figure S1

(A)

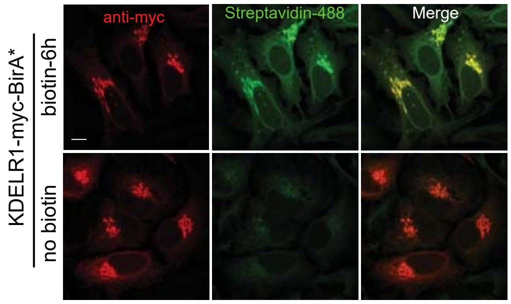

(C)

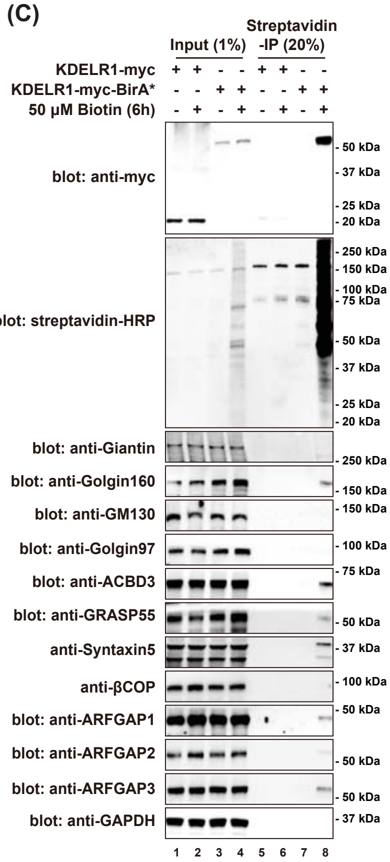

(B)

Protein symbol	peptide number		Sequence coverage (%)		protein abundance ratio	*P* value
	All	Unique	All	Unique		
GRASP55	9	9	26.8	26.8	88.91646	0.001921
Golgin160	34	34	32.4	32.4	62.65546	5.43E-06
ARFGAP3	19	10	45.7	26.9	51.31178	0.002214
Giantin	61	61	25.6	25.6	57.2742	0.000511
ACBD3	24	24	61.7	61.7	11.48065	0.000219
KDELR1	7	4	37.3	27.4	8.362048	0.002843
ARFGAP1	6	6	35.1	35.1	7.696237	0.014567
Syntaxin 5	6	6	22.3	22.3	∞	0.0195
COPG	19	19	35	35	7.435847	0.001571

(D) (E) (F) (G) (H) (I) (J)

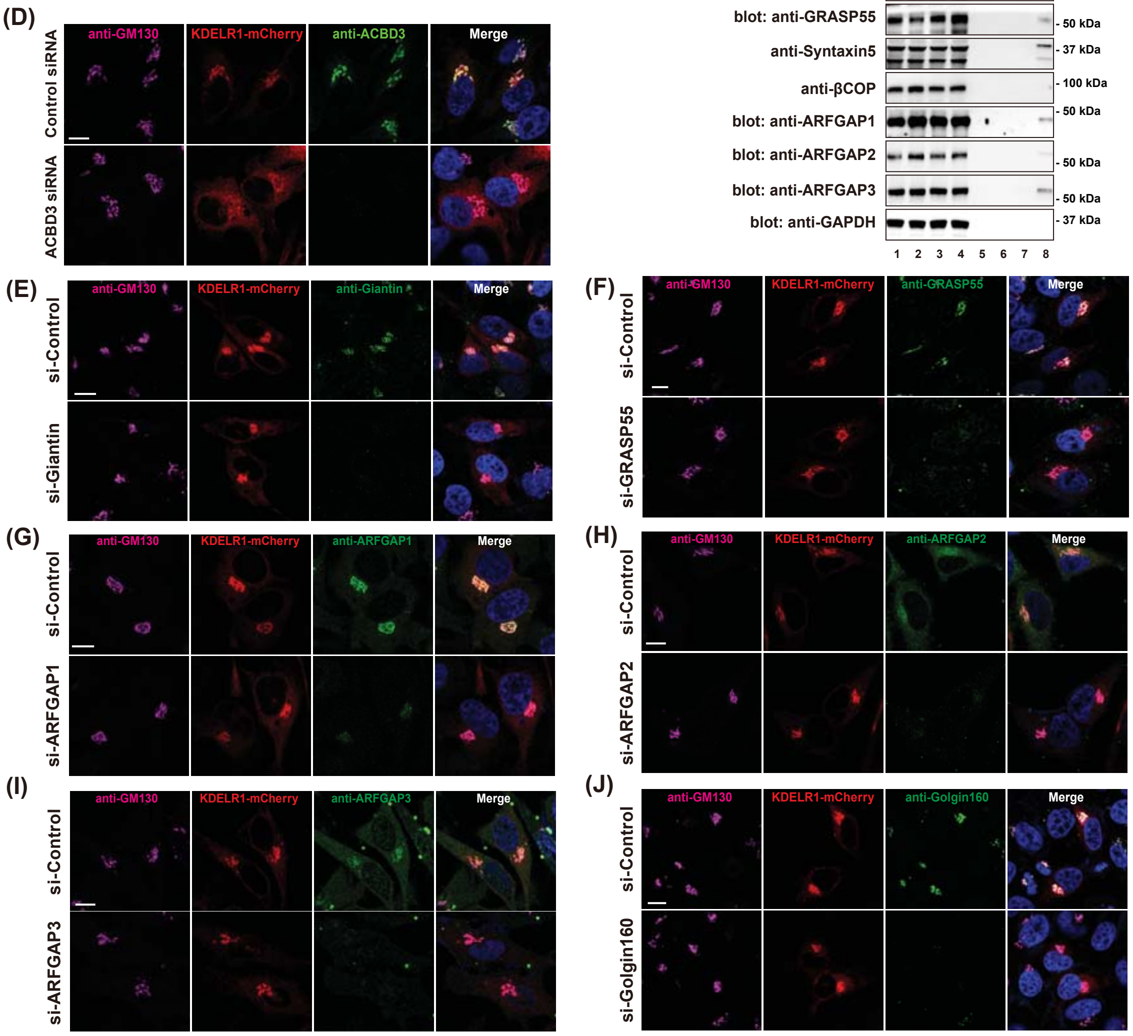

(K)

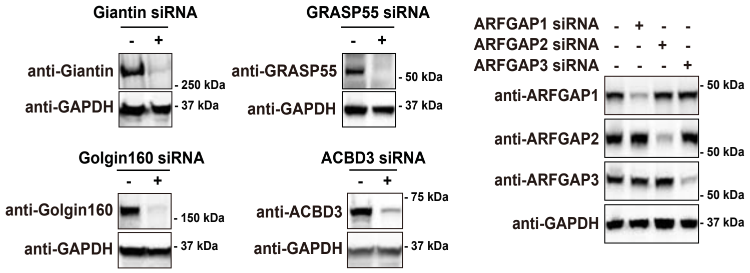

Supplement: Supplementary file 1 — Additional file 1: Figure S1. (A) Characterization of HeLa cells expressing KDELR1 tagged with myc-BirA*. HeLa cells expressing KDELR1-myc-BirA* for 18 hours were grown in media containing biotin (50 μM) for 6 h and double labelled with streptavidin-488 (green) and anti-myc (red). (B) Lists of identified interaction partners in KDELR1-BioID with a number of identified peptides (all and unique) with their sequence coverage. (C) Immunoblotting of KDELR1-BioID samples confirmed biotinylation of the candidate interaction partners identified in mass spectrometry. Anti-GM130 and anti-Golgin97 blots were included as negative controls here. (D - J) Confocal micrographs of HeLa cells expressing KDELR1-mCherry, showing that depletion of Giantin, GRASP55, Golgin160 and ARFGAP1/2/3 do not result in ER re-distribution of KDELR1-mCherry. (K) Western blots showing depletion of Giantin, GRASP55, Golgin160, ACBD3 and ArfGAP1/2/3, respectively, by RNA interference. Scale bars = 10 μm. [file 12915_2021_1137_MOESM1_ESM.pdf]
