# Supplementary figures and images for: ACBD3 modulates KDEL receptor interaction with PKA for its trafficking via tubulovesicular carrier
Source: BMC Biol. 2021 Sep 7;19:194. doi: 10.1186/s12915-021-01137-7 (PMC8424950; doi:10.1186/s12915-021-01137-7)

## Additional File 2: Figure S2

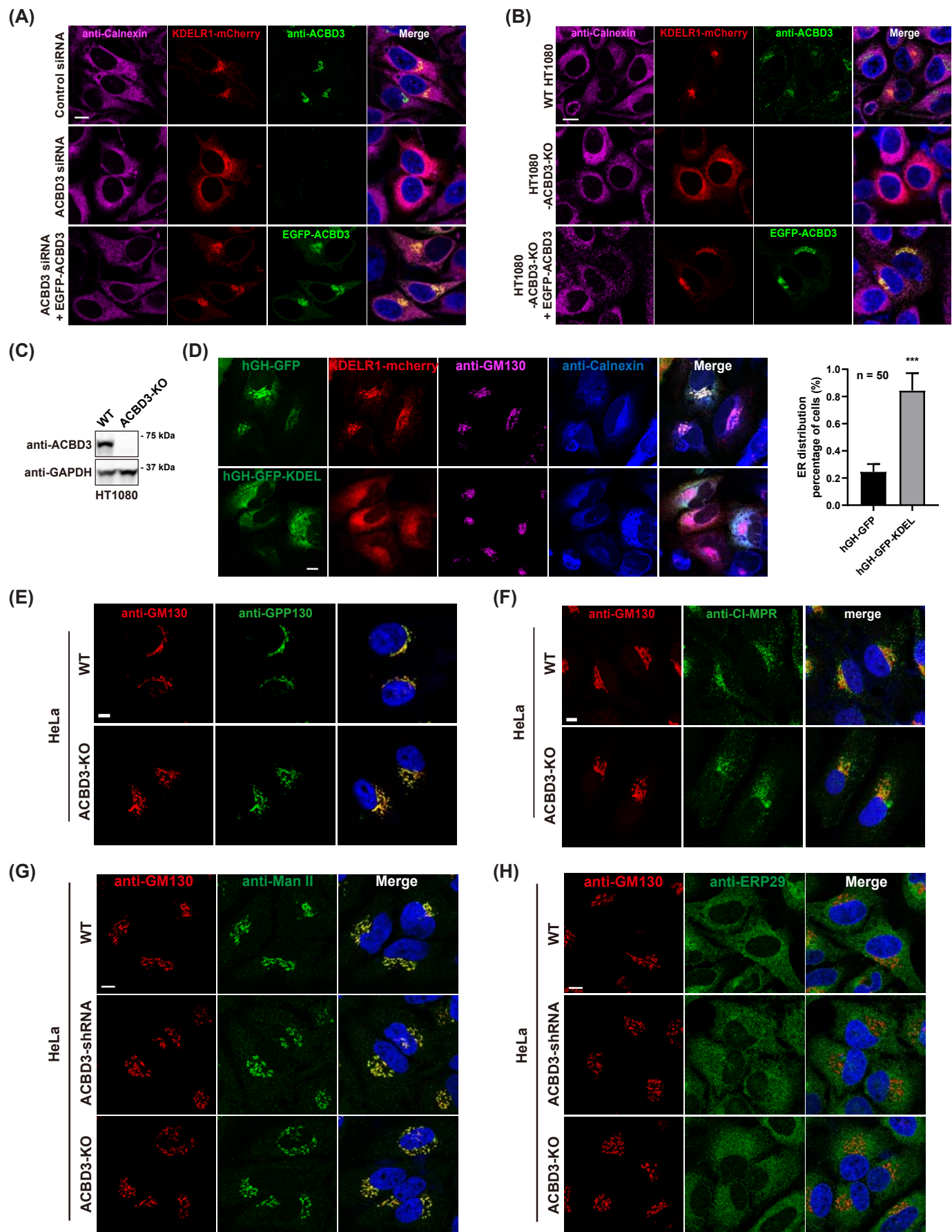

Supplement: Supplementary file 2 — Additional file 2: Figure S2. (A) Confocal micrographs of HeLa cells expressing KDELR1-mCherry showing that depletion of ACBD3 results in re-distribution of KDELR1-mCherry from the Golgi to the ER in vivo (Calnexin; ER marker). Expression of RNAi-resistant form of EGFP-ACBD3 restores perinuclear localization of KDEL receptor in the ACBD3 Knockdown cells. (B-C) ACBD3 knock-out by CRISPR/Cas9 technique in HT1080 cells result in re-distribution of KDELR1-mCherry to the ER. Confocal micrographs of WT and ACBD3-knockout HT1080 cells expressing KDELR1-mCherry showing that knockout of ACBD3 results in relocating KDELR1-mCherry from the Golgi to the ER in vivo. Calnexin was used as an ER-marker in these experiments. (D) Confocal results showing that hGH-GFP-KDEL expression induces exogenously expressed KDELR1-mCherry to re-distribute KDEL receptor to the ER (calnexin-positive compartment). The bar graph shows the summary of the confocal experiments. (***, p < 0.001) (E-F) ACBD3 depletion does not influence Golgi localization of other cycling proteins, such as GPP130 and CI-MPR. (G-H) ACBD3 depletion does not influence Golgi localization of a Golgi resident glycosyltransferase ManII nor secretion of ER-resident chaperone ERP29. scale bar = 10 μm. [file 12915_2021_1137_MOESM2_ESM.pdf]

# Additional File 3: Figure S3

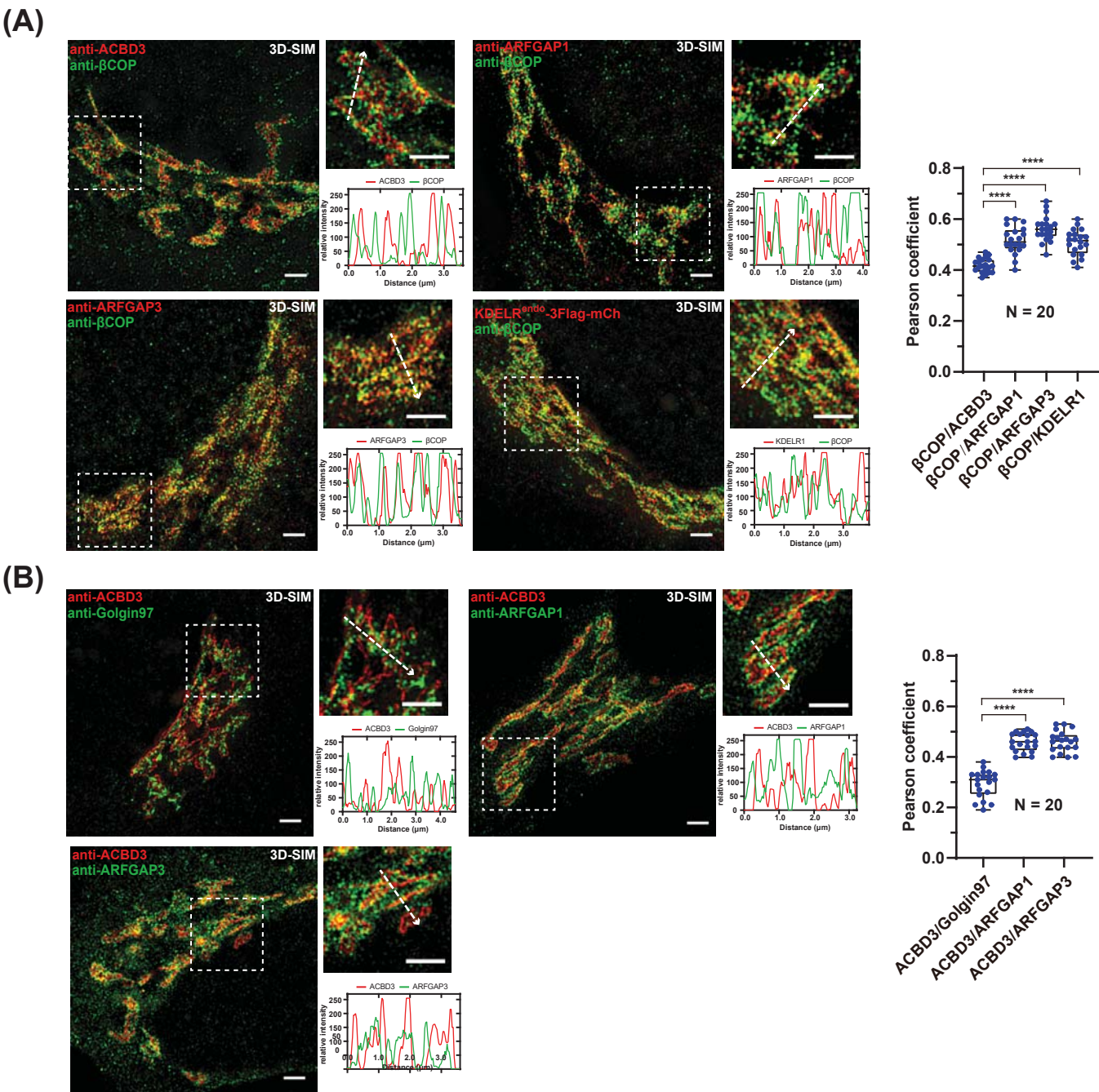

Supplement: Supplementary file 3 — Additional file 3: Figure S3. (A) 3D-SIM images showing that β-COP co-localized most extensively with ArfGAP3 and endogenously tagged KDELR1, followed by ArfGAP1. ACBD3 and β-COP didn’t show a significant overlap. Line profiles through regions of interest were analyzed by Fiji. Scale bars = 2 μm. Co-localization (Pearson’s R) was determined and subjected to two-tailed, unpaired t tests (n = 20 cells/combination, mean and SD, ****, p < 0.0001). (B) 3D-SIM images showing moderate co-localization between endogenous ACBD3 and endogenous ARFGAP1/3. No co-localization between endogenous ACBD3 and Golgin97, which serves as a negative control. Line profiles through regions of interest were analyzed by Fiji. Scale bars = 2 μm. Co-localization (Pearson’s R) was determined and subjected to two-tailed, unpaired t tests (n = 20 cells/combination, mean and SD, ****, p < 0.0001). [file 12915_2021_1137_MOESM3_ESM.pdf]

Additional File 4:  
Figure S4

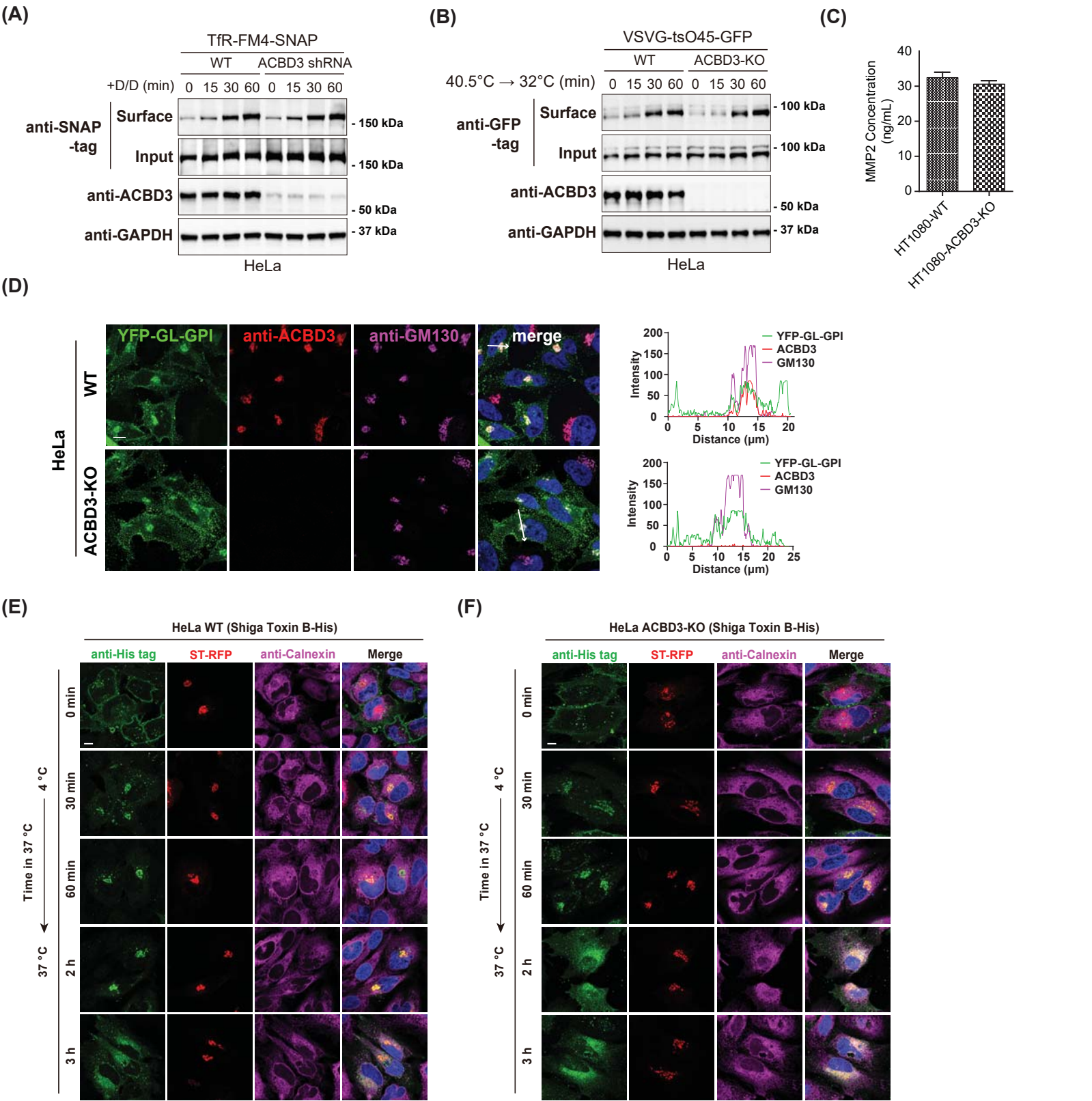

Supplement: Supplementary file 4 — Additional file 4: Figure S4. Anterograde transport of secretory cargo proteins is not significantly altered in ACBD3-depleted cells. In order to investigate whether ACBD3 depletion might have affected anterograde transport between the ER and the Golgi, secretion of three different cargo proteins was tested, including TfR-RM4-SNAP (A), VSVG-tsO45-GFP (B), endogenous MMP-2 (C) and YFP-GL-GPI (D). (A-B) Briefly, plasmids encoding the indicated constructs were transiently transfected into control cells or ACBD3-depleted HeLa cells for 18 hours. Cells were then treated with cycloheximide for 2 hours, prior to induction of synchronized protein secretion by shifting temperature from 40.5 to 32 °C. (VSVG-tsO45-GFP) or treatment with D/D solubilizer drug (TfR-FM4-SNAP) for the indicated times. At the indicated timepoints, the cells were placed on ice and subjected to surface biotinylation using sulfo-NHS-LC-biotin for 30 min. The cells were then lysed, subjected to pulldown with streptavidin-agarose and analyzed by western blot. (C) For MMP2 measurement, the conditioned media from control HT1080 or ACBD3-KO HT1080 cells were collected after 18 hours incubation and added to Total MMP2 Quantikine ELISA kit for quantification, as described in the methods. (D) After 18 hrs transfection of YFP-GL-GPI, HeLa WT and ACBD3-KO cells were stained for indicated antibodies and then examined by confocal microscopy. Line profiles through regions of interest were analyzed by Fiji. (Scale bars = 10 μm) (E-F) HeLa-WT or HeLa-ACBD3-KO cells were transfected with sialyltransferase-RFP (ST-RFP, a Golgi marker) and His-tagged Shiga toxin B fragment (2.5 mg/ml final concentration in DMEM+1%FBS) was added to cells for 45min at 4°C. After the withdrawal of unbound toxin by washing for three times in ice-cold PBS, cells were incubated with DMEM+10%FBS at 37°C for indicated time points. Then cells were stained using anti-His-tag and anti-calnexin (as an ER marker) antibodies. The results show that pl [file 12915_2021_1137_MOESM4_ESM.pdf]
