# Supplementary material for: ACBD3 modulates KDEL receptor interaction with PKA for its trafficking via tubulovesicular carrier
Source: BMC Biol. 2021 Sep 7;19:194. doi: 10.1186/s12915-021-01137-7 (PMC8424950; doi:10.1186/s12915-021-01137-7)

Additional File 5: unprocessed images of western blots

Unprocessed western blots in main and supplementary figures.
Some blots were cut into several pieces and incubated with different antibodies.

Unprocessed blots of Figure 1C

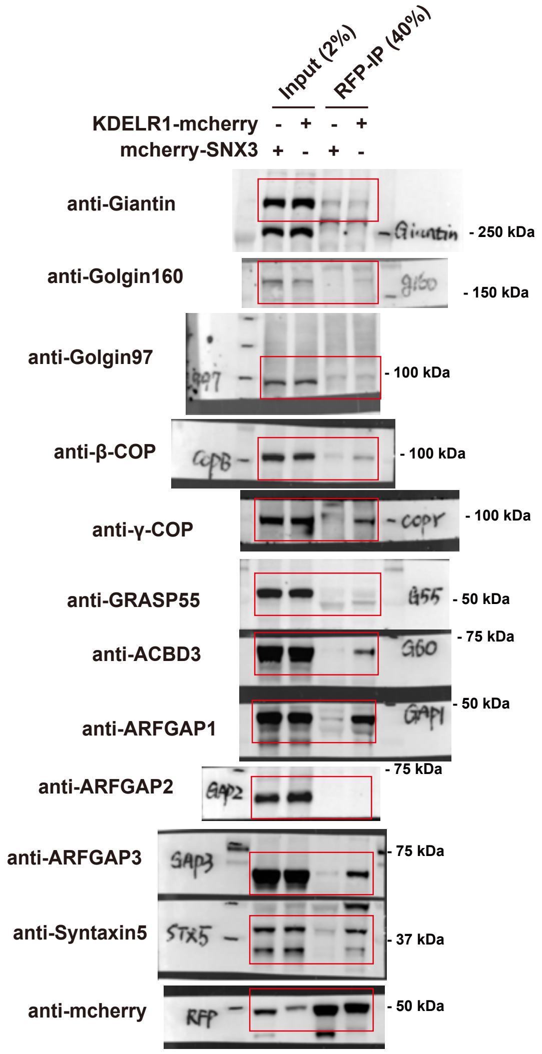

Unprocessed blots of Figure 2G

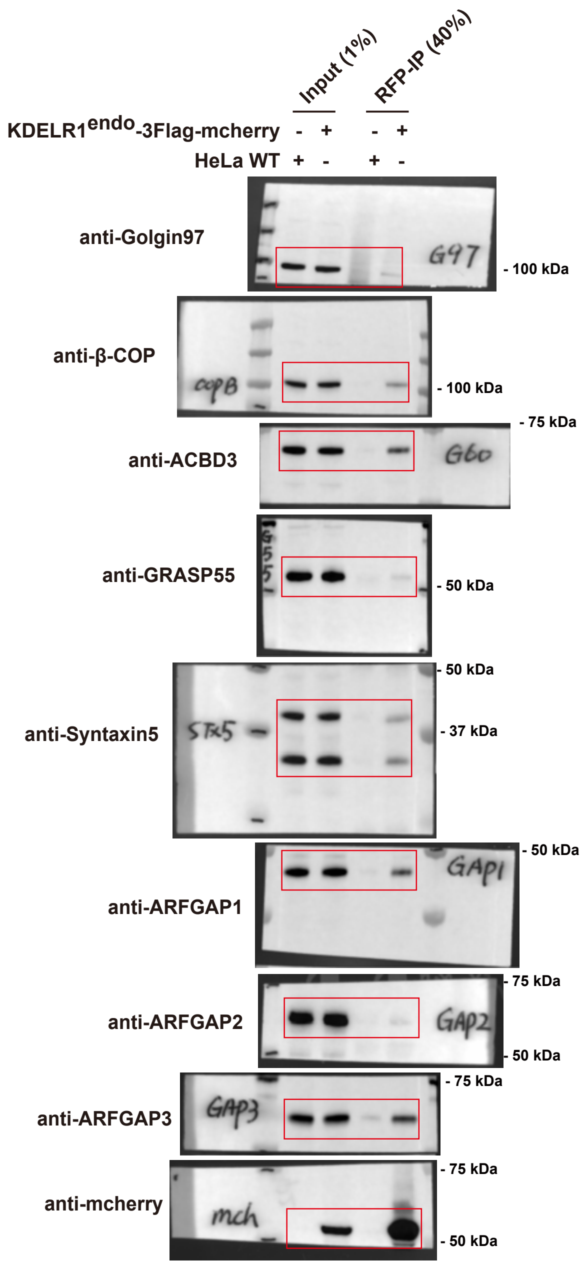

Unprocessed blots of Figure 3D

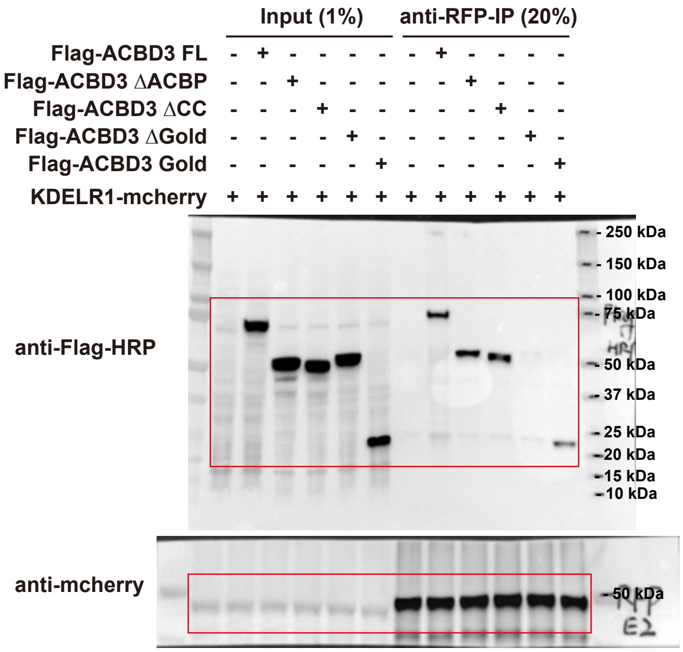

Unprocessed blots of Figure 4E

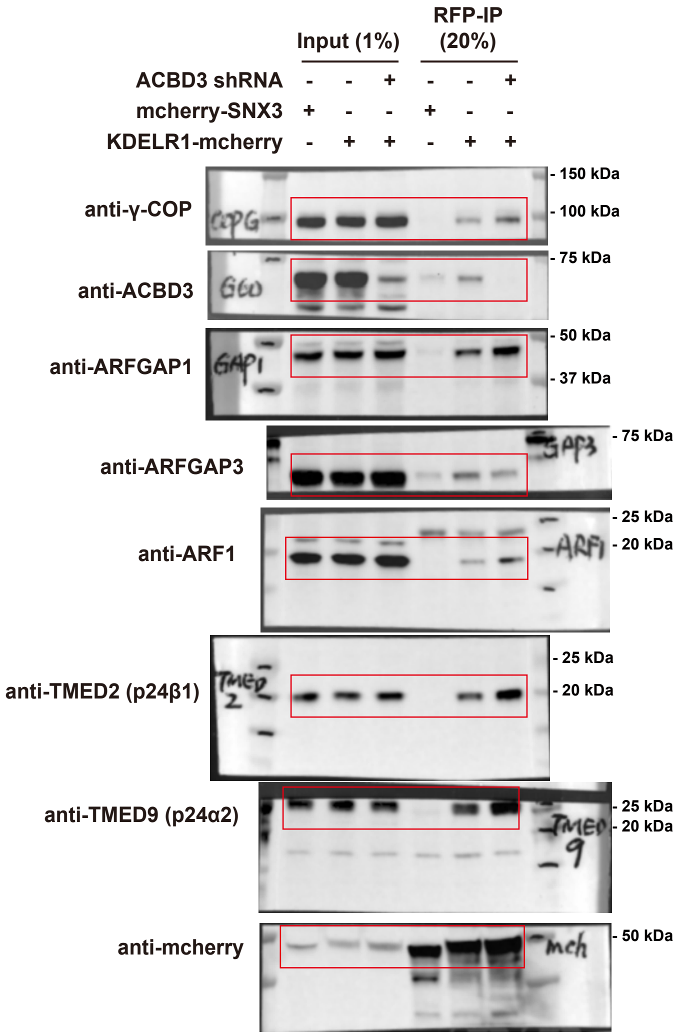

Unprocessed blots of Figure 5A

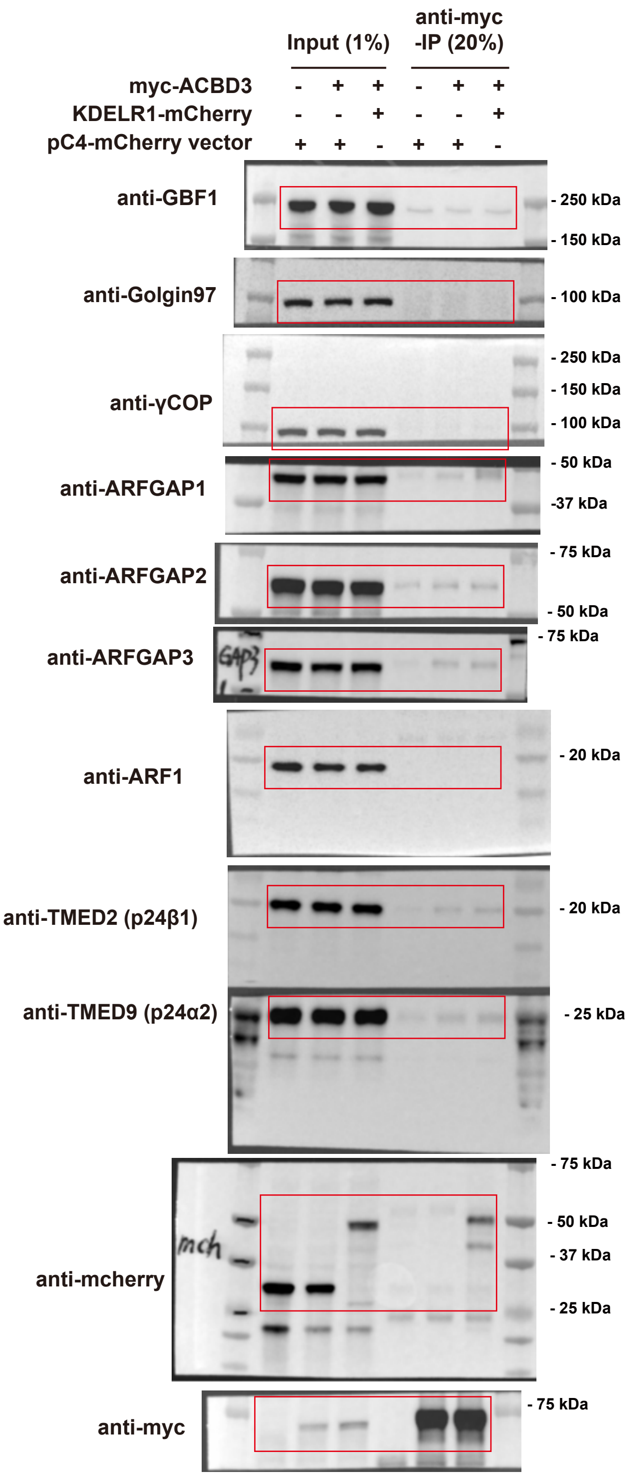

Unprocessed blots of Figure 5B&C

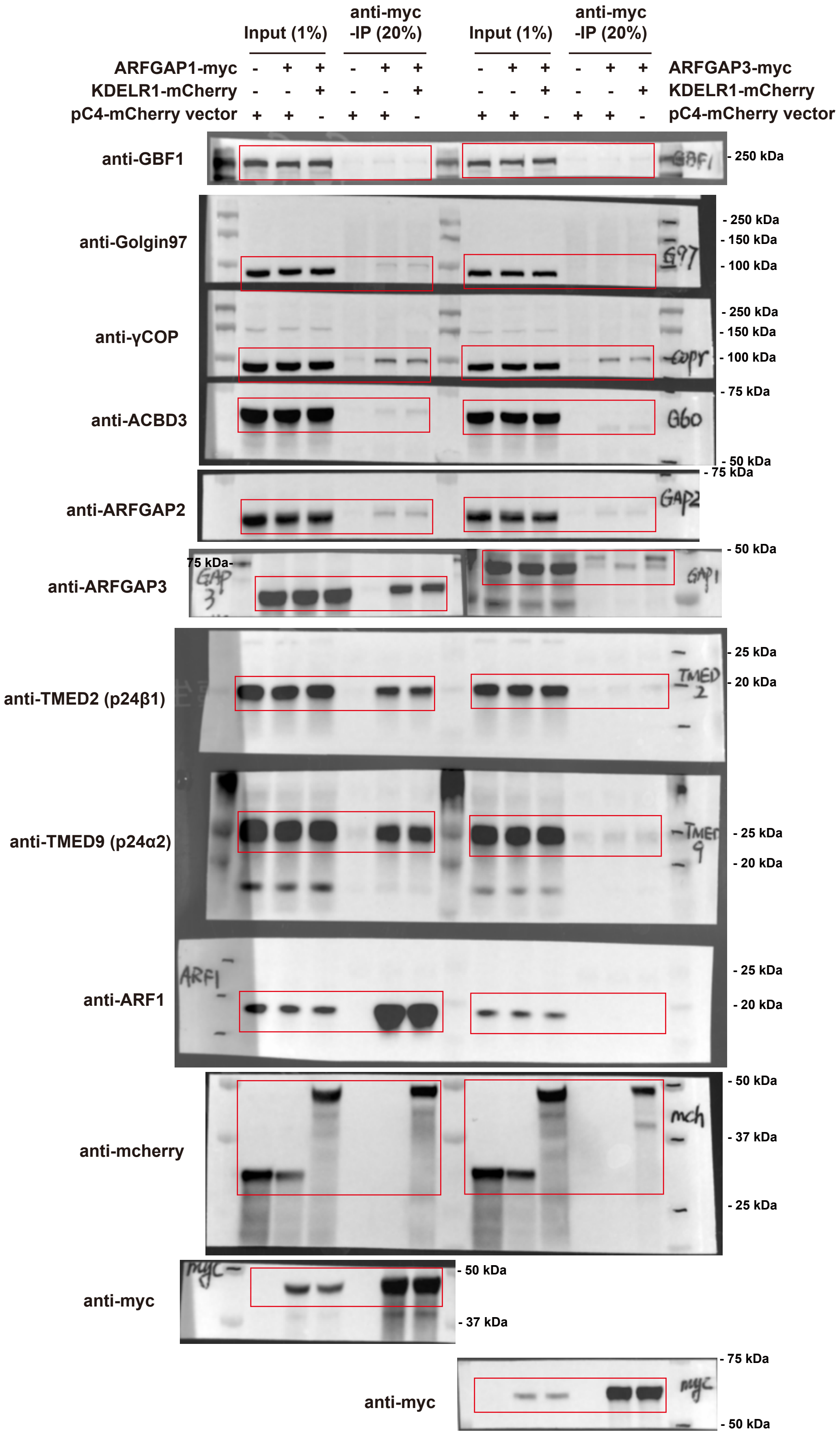

Unprocessed blots of Figure 5F

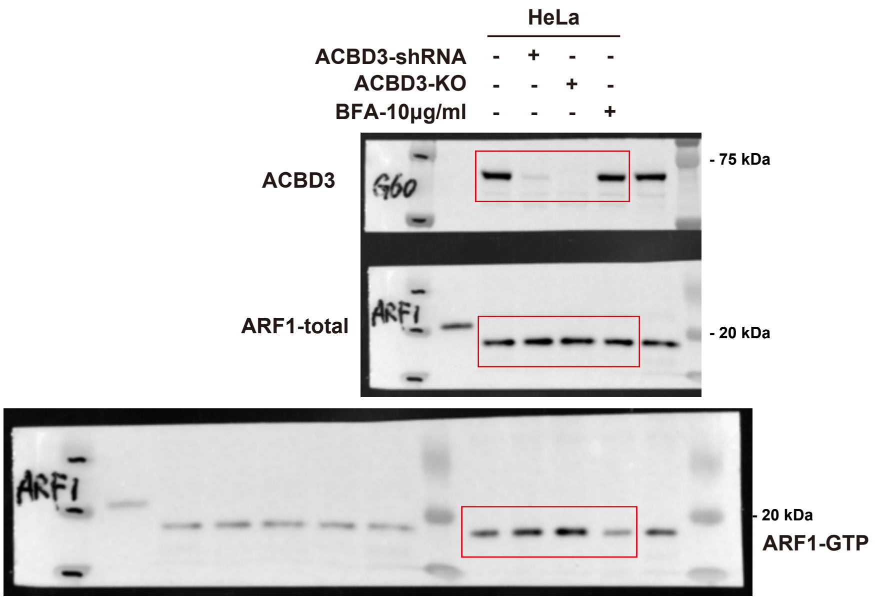

Unprocessed blots of Figure 5I

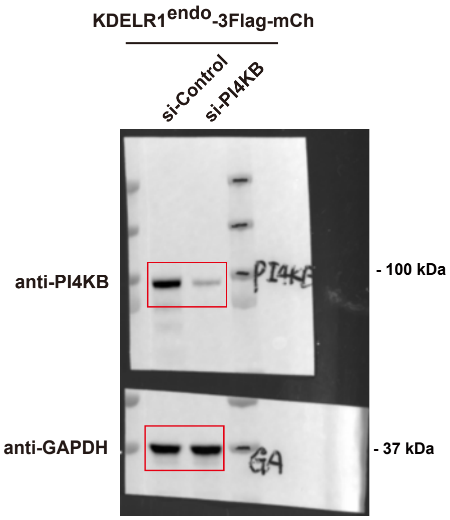

Unprocessed blots of Figure 6A

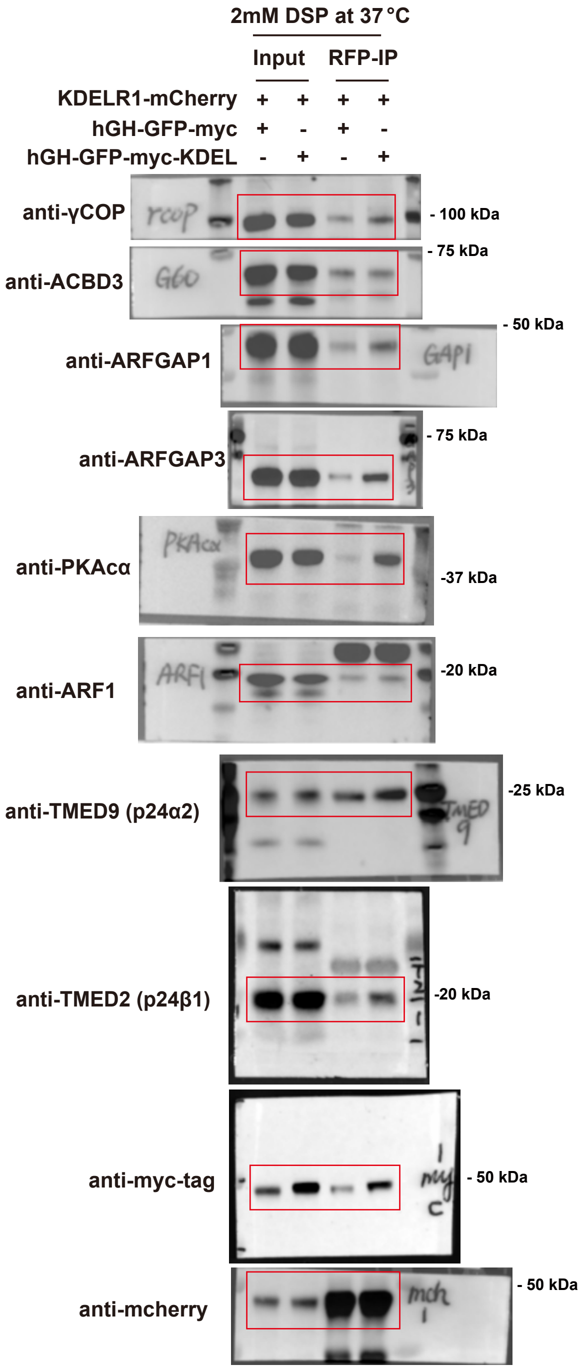

Unprocessed blots of Figure 6B

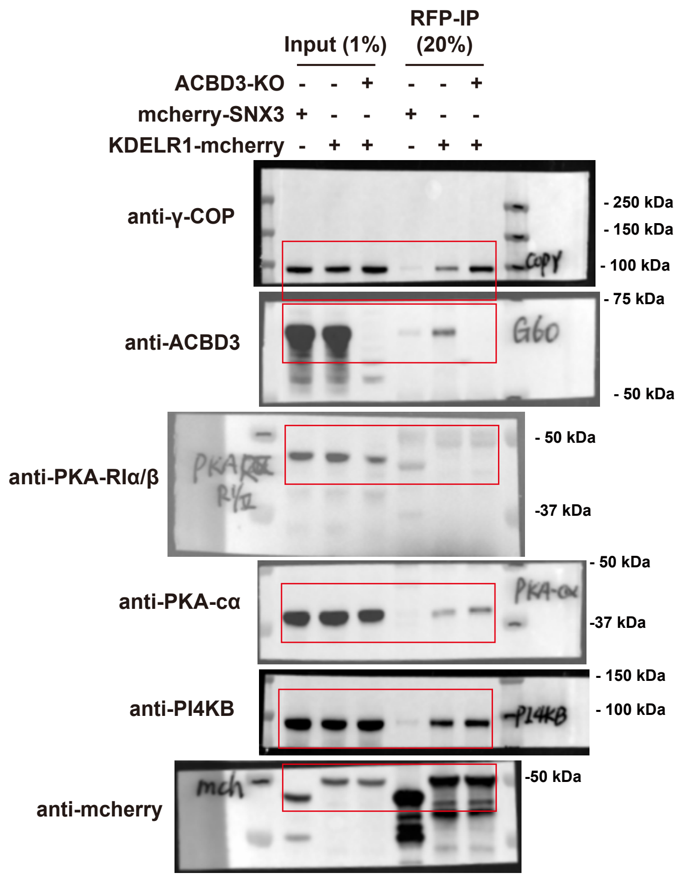

Unprocessed blots of Figure 6C

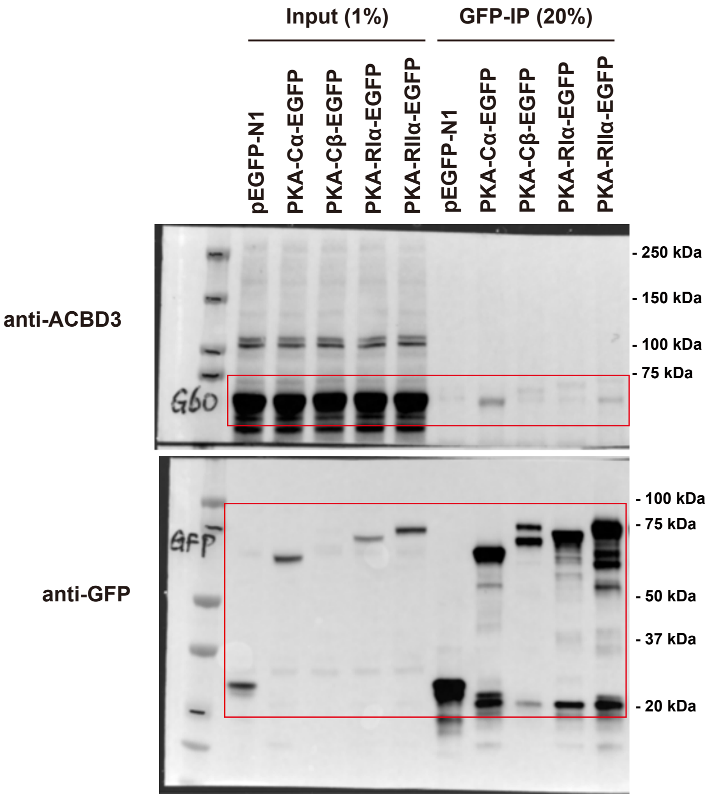

Unprocessed blots of Figure 6D

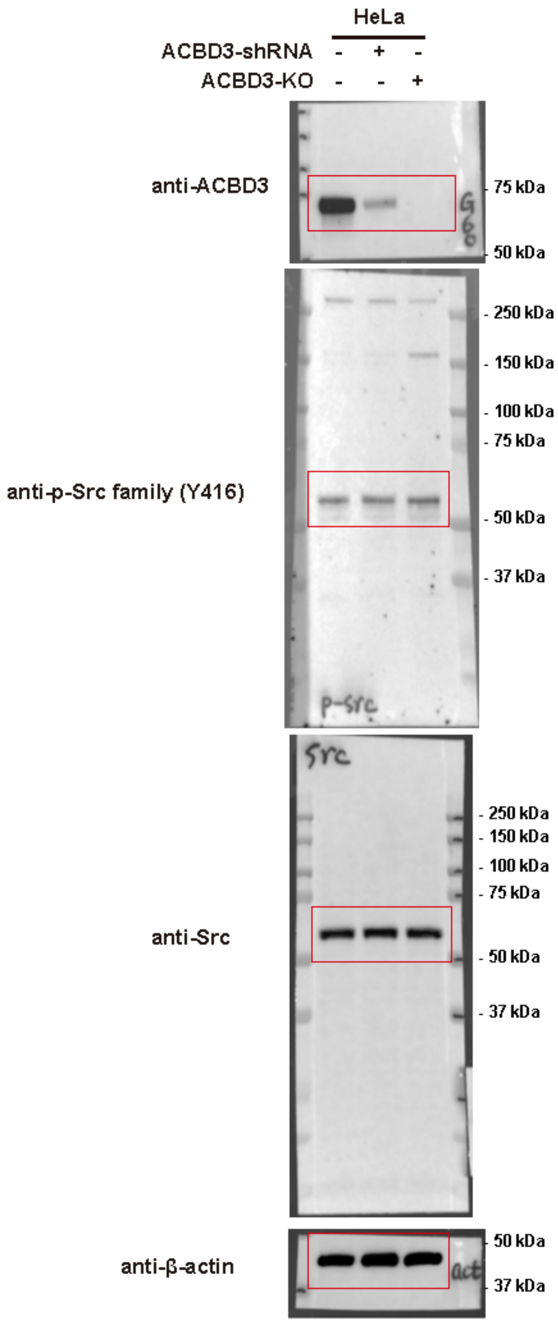

Unprocessed blots of Figure 6F

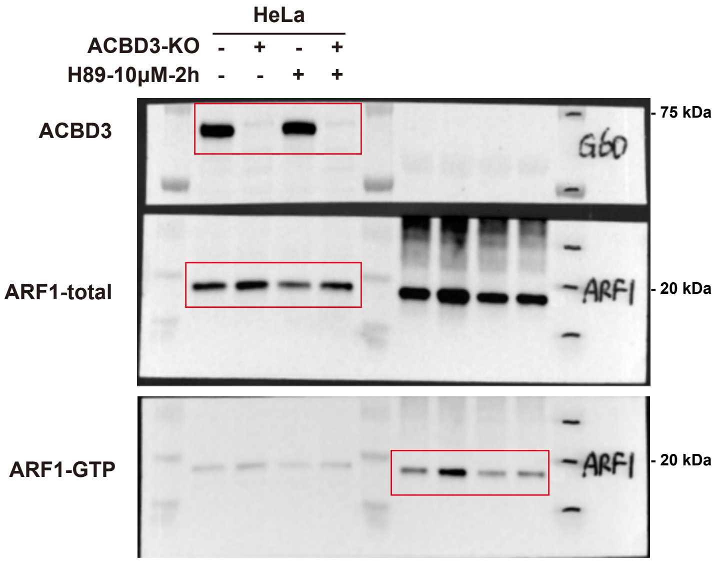

Unprocessed blots of Figure 7A

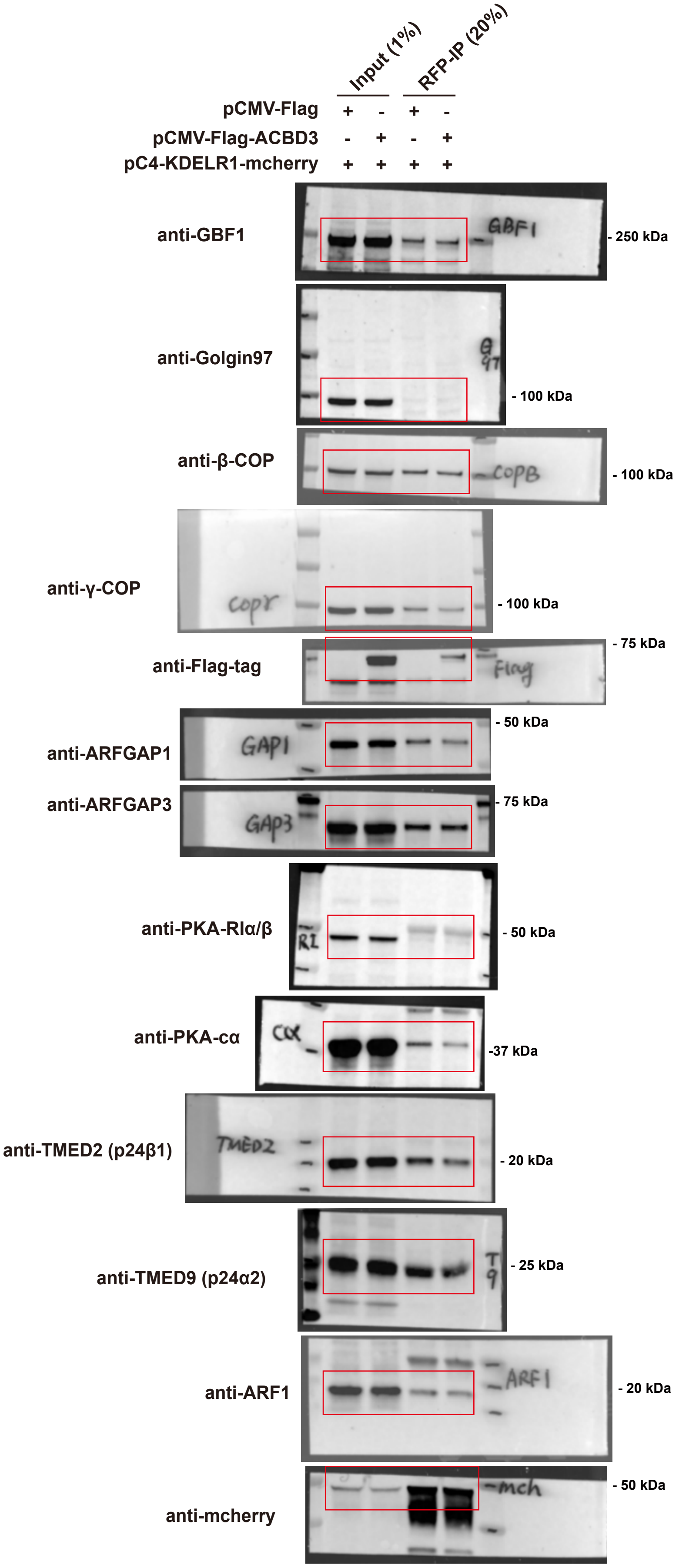

Unprocessed blots of Figure 7H

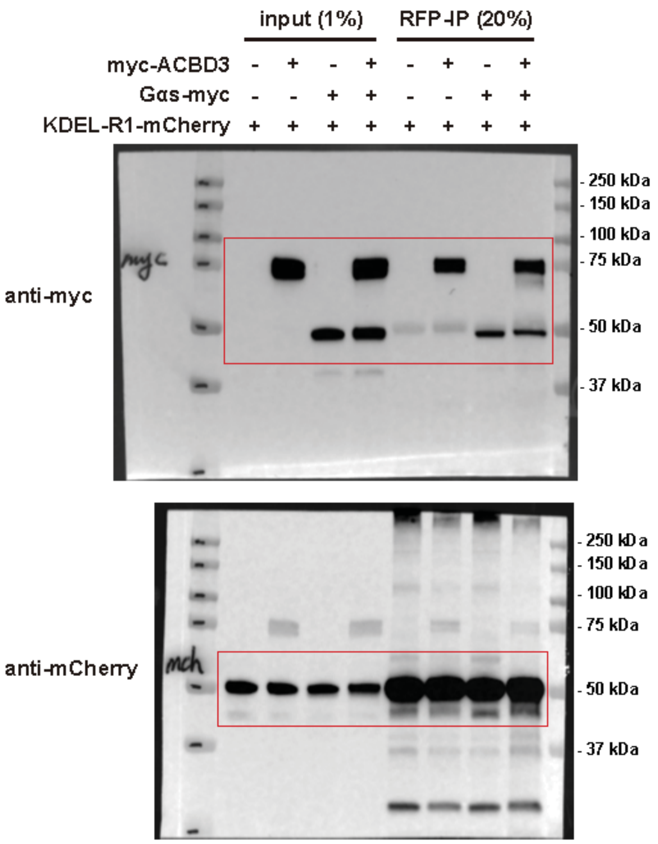

Unprocessed blots of figure S1C

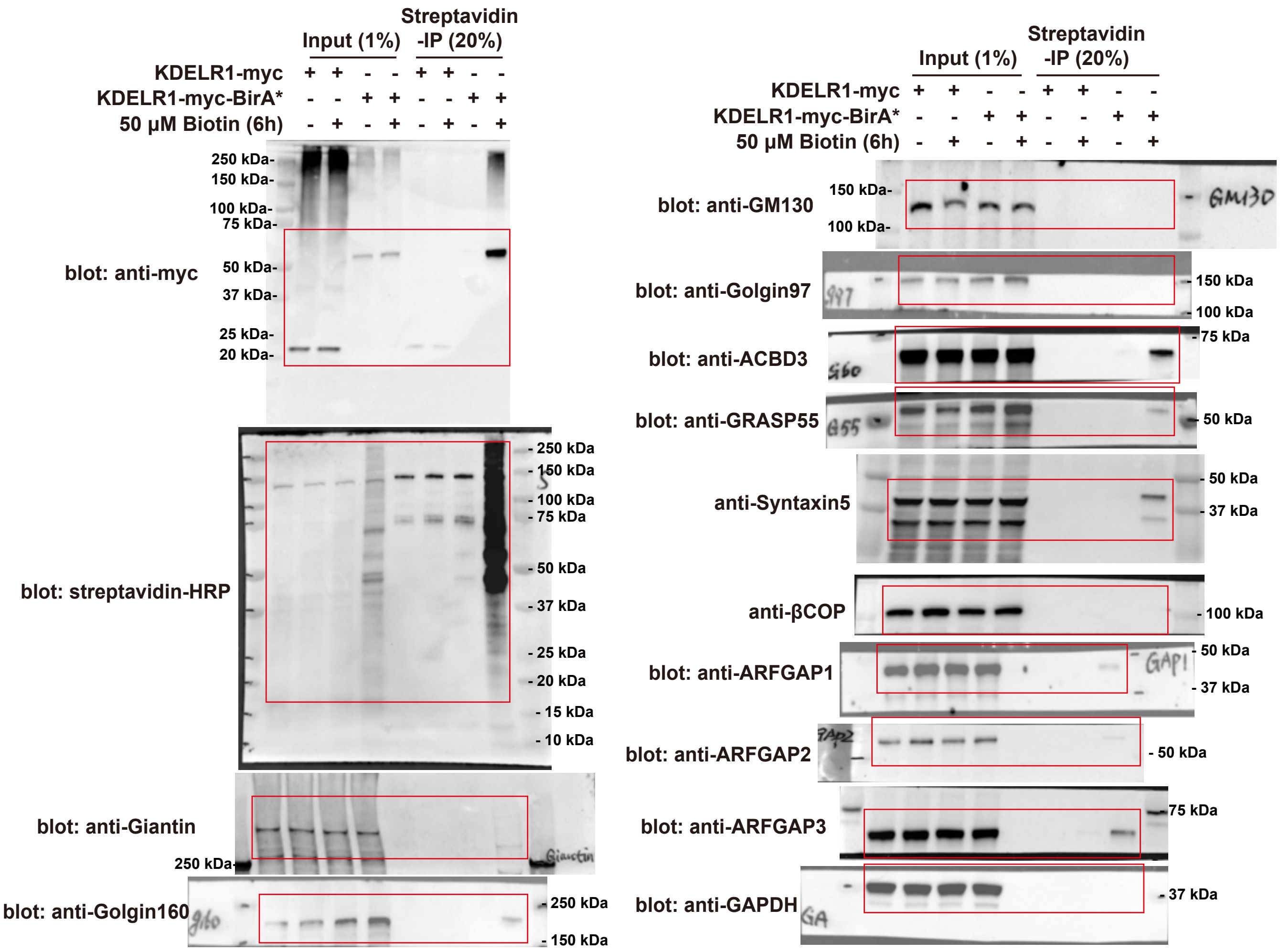

Unprocessed blots of figure S1K

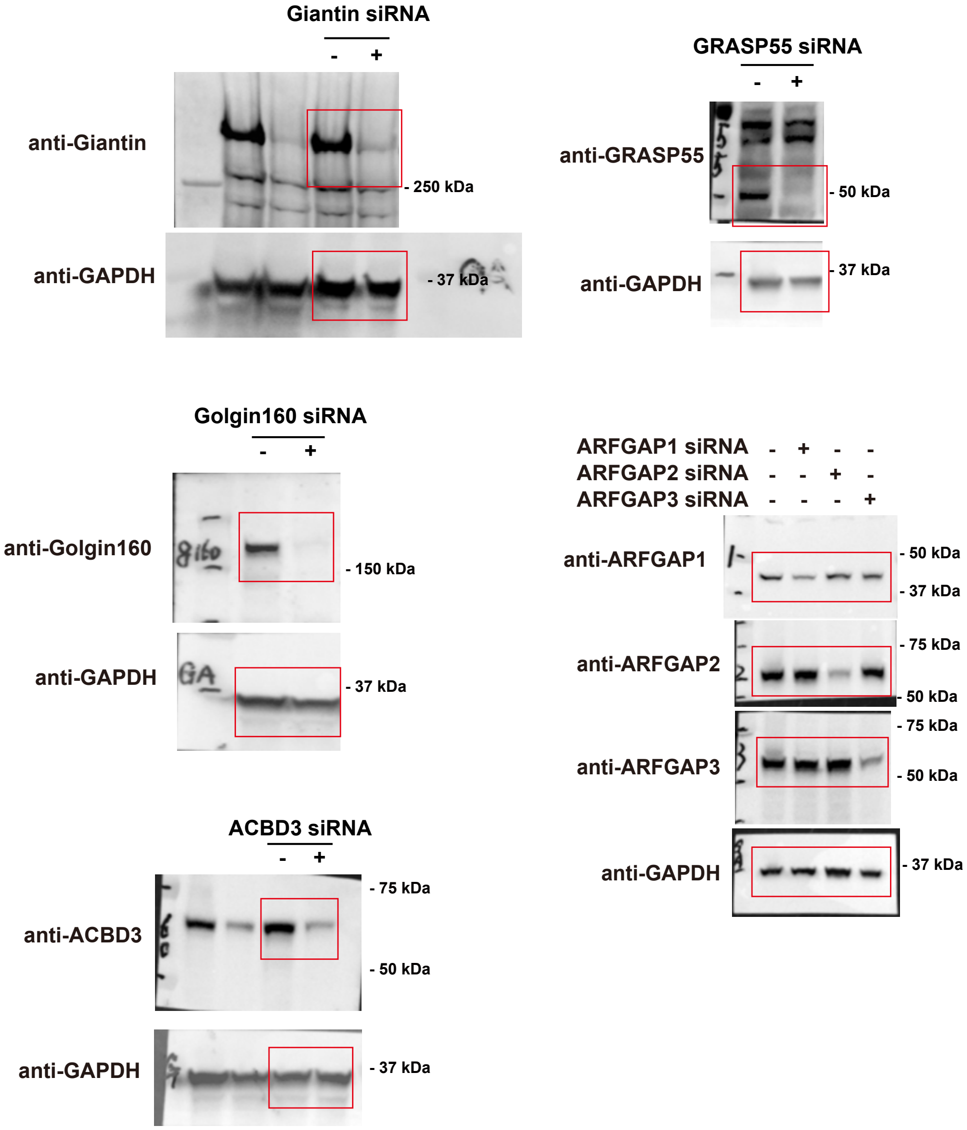

Unprocessed blots of figure S2C

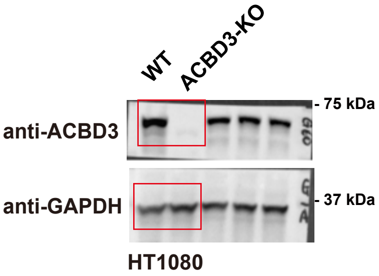

Unprocessed blots of Figure S4

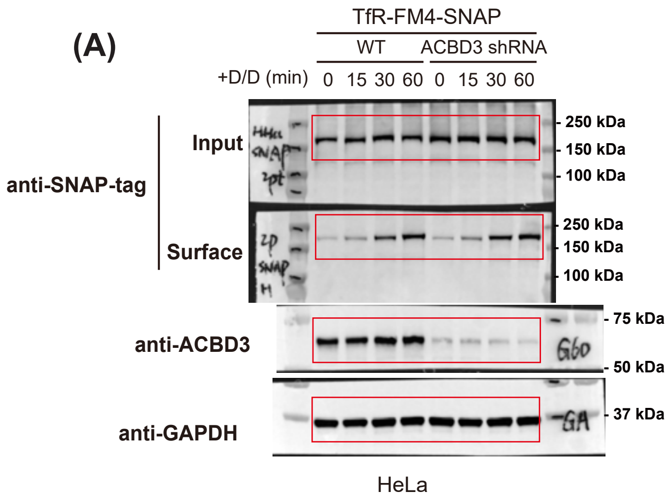

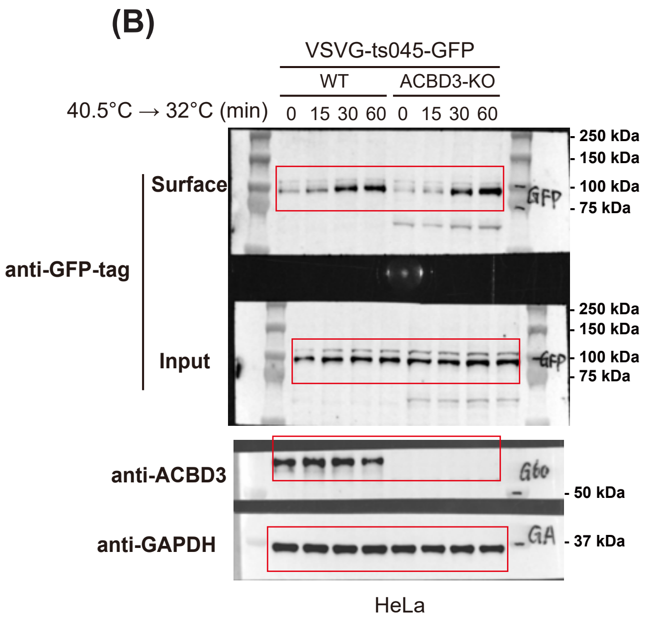

Supplement: Supplementary file 5 — Additional file 5. Raw-data-Western blotting. [file 12915_2021_1137_MOESM5_ESM.pdf]
